# A *Drosophila* model of oral peptide therapeutics for adult intestinal stem cell tumors

**DOI:** 10.1242/dmm.044420

**Published:** 2020-07-24

**Authors:** Anjali Bajpai, Taushif Ahmad Quazi, Hong-Wen Tang, Nishat Manzar, Virender Singh, Ashwani Thakur, Bushra Ateeq, Norbert Perrimon, Pradip Sinha

**Affiliations:** 1 Biological Sciences and Bioengineering, Indian Institute of Technology Kanpur, Kanpur 208016, India; 2 Department of Genetics, Blavatnik Institute, Harvard Medical School, Boston, MA 02115, USA; 3 Department of Physiology and Biophysics, Case Western Reserve University, Cleveland, OH 44106, USA; 4 Howard Hughes Medical Institute, Boston, MA 02115, USA

**Keywords:** *Drosophila*, Peptide therapeutic, Yki, Intestinal stem cells, Integrin signaling

## Abstract

Peptide therapeutics, unlike small-molecule drugs, display crucial advantages of target specificity and the ability to block large interacting interfaces, such as those of transcription factors. The transcription co-factor of the Hippo pathway, YAP/Yorkie (Yki), has been implicated in many cancers, and is dependent on its interaction with the DNA-binding TEAD/Sd proteins via a large Ω-loop. In addition, the mammalian vestigial-like (VGLL) proteins, specifically their TONDU domain, competitively inhibit YAP-TEAD interaction, resulting in arrest of tumor growth. Here, we show that overexpression of the TONDU peptide or its oral uptake leads to suppression of Yki-driven intestinal stem cell tumors in the adult *Drosophila* midgut. In addition, comparative proteomic analyses of peptide-treated and untreated tumors, together with chromatin immunoprecipitation analysis, reveal that integrin pathway members are part of the Yki-oncogenic network. Collectively, our findings establish *Drosophila* as a reliable *in vivo* platform to screen for cancer oral therapeutic peptides and reveal a tumor suppressive role for integrins in Yki-driven tumors.

This article has an associated First Person interview with the first author of the paper.

## Abstract (precis)

**Summary:** We demonstrate that, when fed to flies, TONDU peptide suppresses Yki-driven intestinal stem cell (ISC) tumors, and identify integrins as essential components of ISC tumorigenesis.

## INTRODUCTION

*Drosophila* has emerged as an effective tumor model for the screening of small-molecule therapeutics (Dar et al., 2012; Khoo et al., 2013; Markstein et al., 2014; Bangi et al., 2016). Whereas cancer-promoting misregulated kinases are amenable to inhibition by small molecules, others, such as transcription factors (TFs) and co-factors, are largely considered undruggable (Bhagwat and Vakoc, 2015; Lambert et al., 2018). In this regard, peptides are particularly attractive as therapeutic molecules (Lau and Dunn, 2018; Drucker, 2019) because of their high selectivity, improved tolerance and ability to target large interacting interfaces (Furet et al., 2019). While most peptide therapeutics require parenteral injection, their oral delivery is highly desirable; indeed, currently a few orally derived therapeutic peptides are being tested in clinical trials (Drucker, 2019).

The proto-oncogene Yes-associated protein [YAP; Yorkie (Yki) in *Drosophila*] – the transcription co-factor of the Hippo pathway – interacts with its DNA-binding partner, transcriptional enhanced associate domain 1-4 [TEAD1-4; Scalloped (Sd) in *Drosophila*] (Wu et al., 2008), and is implicated in cancers (Zanconato et al., 2016). YAP binds to TEAD via an unusually large interface, the Ω-loop (Pobbati et al., 2012; Furet et al., 2019), which lacks a defined binding pocket, making it an unlikely target of inhibition by small molecules. TEAD proteins also bind to other transcriptional co-factors, such as the vestigial-like (VGLL1-4) proteins that display a highly conserved 26 amino acid TONDU domain (Pobbati et al., 2012; Koontz et al., 2013). VGLL4 competitively inhibits binding of YAP and TEAD, thereby acting as a tumor suppressor (Zhang et al., 2014). Interestingly, a synthetic peptide analog of the TONDU domain of VGLL4 was found to inhibit gastric cancer growth (Jiao et al., 2014) in a mouse xenograft model.

Similar to the mammalian VGLL-TEAD-YAP partnership, *Drosophila* TONDU-containing proteins, such as Vestigial (Vg) and Tondu-domain-containing Growth Inhibitor (Tgi) interact with Sd and Yki (Guo et al., 2013; Koontz et al., 2013). Sd, when not bound to Yki, interacts with the ubiquitously expressed Tgi via the Tgi TONDU domain. The conserved interaction between Vg/Tgi with Sd-Yki in *Drosophila* therefore makes the fly a relevant platform to screen for large-molecule inhibitors of YAP-TEAD interaction. Here, we used the adult *Drosophila* gut – which displays Sd-dependent Yki activity for intestinal stem cell (ISC) homeostasis (Jin et al., 2013) – to test whether a TONDU peptide can suppress ISC tumors triggered by gain of an activated form of Yki (Kwon et al., 2015; Song et al., 2019). We show that ISC tumors in the adult midgut induced by gain of activated Yki are suppressed by feeding TONDU peptide-supplemented food. Further, comparative proteome analysis and genetic tests reveal that integrin pathway members are part of the Yki-oncogenic network. Altogether, our results establish our *Drosophila* ISC tumor model as a reliable platform for screening therapeutic peptides with the added advantage of rapid resolution of the mechanistic underpinning of tumor suppression.

## RESULTS

### Genetic suppression of Yki-driven ISC tumor growth by the TONDU peptide

**Fig. 1.**
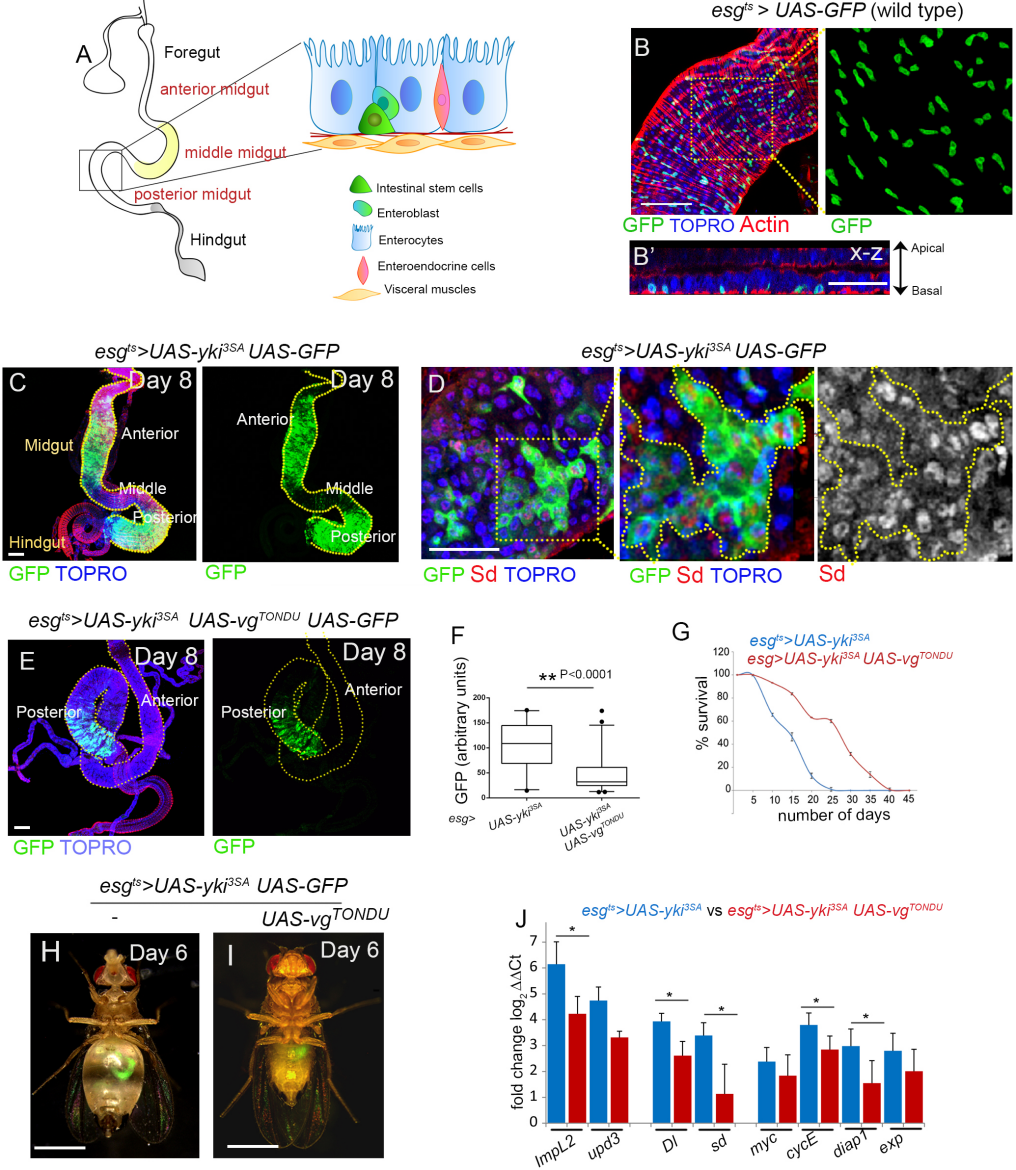
**Expression of the TONDU peptide inhibits Yki-driven ISC tumors.** (A) Schematic representation depicting the different cell types in the adult *Drosophila* gut. (B,B′) *esg^ts^>UAS-GFP* labels ISCs in the *Drosophila* midgut. (B) ISCs (marked by GFP) are interspersed throughout the gut. Overlying muscles are marked with F-Actin (red). (B′) X-Z section displaying basally located ISCs (GFP). (C) *esg^ts^>yki^3SA^UAS-GFP* gut shows an increase in ISC numbers. (D) *esg^ts^>yki^3SA^UAS-GFP* tumors show increase in Sd level. (E) Decrease in ISCs (marked by GFP) in the anterior and posterior midgut of *esg^ts^>yki^3SA^ UAS-vg^TONDU^* flies that co-express the TONDU peptide. (F) Quantification of GFP in TONDU-expressing and non-expressing *esg^ts^>yki^3SA^*guts. Box plots indicate the median (horizontal lines), 25th and 75th percentiles (box), and 2.5 to 97.5 percentile range (whiskers). Outliers are displayed as filled circles. Significance displayed as *P*-value, for unpaired Student's *t*-test. (G) Increase in survival of *esg^ts^>yki^3SA^UAS-vg^TONDU^* flies compared to *esg^ts^>yki^3SA^* (*n*=50 each genotype). (H) Abdominal bloating in *esg^ts^>yki^3SA^UAS-GFP* flies as seen on day 6 after tumor induction (*n*=19/25 are bloated). (I) *esg^ts^>yki^3SA^ UAS-vg^TONDU^ UAS-GFP* flies display delay in bloating (*n*=14/25 are not bloated) as seen on day 6. (J) qPCR displaying the decrease in mRNA levels of candidate genes in TONDU-expressing flies. Data presented as mean±s.e.; *P≤0.025 for Student's *t*-test. Scale bars: 100 µm (B,C,E), 50 µm (B′,D), 1 mm (H,I).

The *Drosophila* gut closely resembles the mammalian gut and is divided into the foregut, midgut and hindgut (Guo et al., 2016). The midgut makes up most of the gut and contains three cell types: differentiated enterocytes, entero-endocrine cells and ISCs (Fig. 1A,B). Expression of a phosphorylation-defective and therefore constitutively active form of Yki in the ISCs (*esg-Gal4 Gal80^ts^>UAS-yki^3SA^*, referred to as *esg^ts^>yki^3SA^*) results in gut stem cell tumors (Kwon et al., 2015) (Fig. 1C; Fig. S1A-D). Yki gut tumor-bearing flies display a systemic wasting syndrome (Kwon et al., 2015) (Fig. 1H; Fig. S2A) and display elevated levels of the insulin antagonist *ImpL2* (Fig. 1J) (Kwon et al., 2015), in addition to canonical Yki targets (Fig. 1J) that include Sd (Fig. 1D,J), the DNA-binding partner of Yki (Wu et al., 2008).

Since the TONDU-containing proteins Vg (Khan et al., 2013) and Tgi (Guo et al., 2013; Koontz et al., 2013) can inhibit Yki-regulated growth by competing for Sd, we tested whether co-expression of TONDU peptide alone (CVVFTNYSGDTASQVDEHFSRALNY) in ISCs with gain of Yki (*esg^ts^*>*yki^3SA^ UAS-vg^TONDU^*) would inhibit Yki-driven ISC tumor growth. Indeed, a striking inhibition in ISC tumors (Fig. 1E,F), with an accompanying loss of proliferation (Fig. S2C-E), was seen under this condition. In addition, these flies showed improved life span (Fig. 1G) and a delay in the onset of tumor-associated wasting phenotypes (Fig. 1H,I; Fig. S2B,F,G), with a concomitant decrease in expression of *Impl2* (Fig. 1J), a hallmark of *esg^ts^>yki^3SA^* tumors (Kwon et al., 2015). By contrast, overexpression of the TONDU peptide alone in ISCs (*esg^ts^>vg^TONDU^*) did not affect the number of ISCs (Fig. S2H,I). Altogether, these results reveal that Yki-driven ISC tumors are suppressed upon co-expression of the TONDU peptide, with an accompanying delay in the onset of tumor-associated syndromes.

### Oral uptake of synthetic TONDU peptide inhibits Yki-driven ISC tumors

**Fig. 2.**
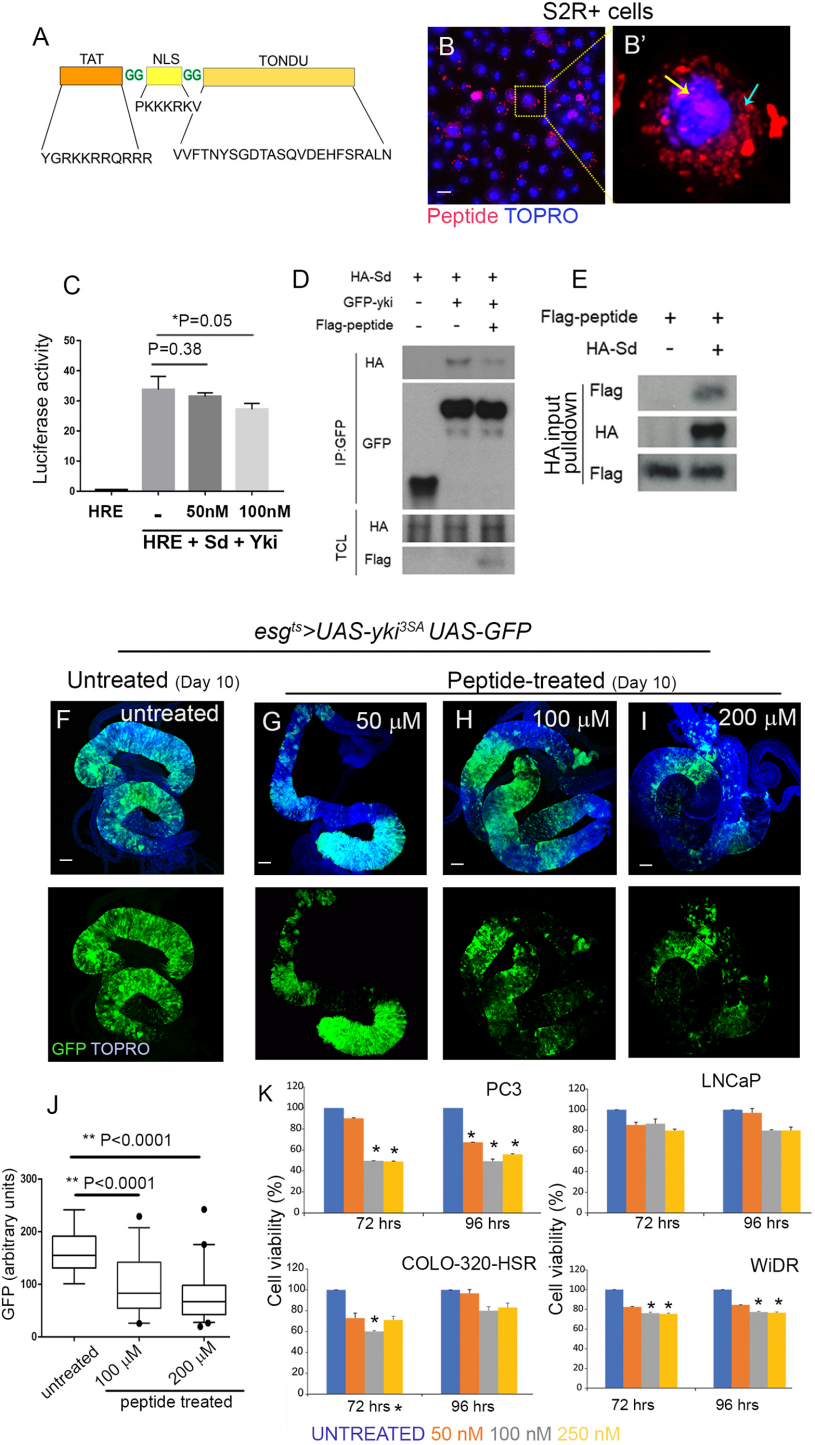
**Synthetic TONDU peptide inhibits Yki-driven ISC tumors.** (A) Representation of the synthetic TONDU peptide. (B,B′) Nuclear localization of fluorescent-tagged (red) TONDU peptide in S2R+ cells. (B′) Magnified view of the boxed area in B. TONDU peptide (red) in the nucleus (yellow arrow) and cytoplasm (cyan arrow). (C) HRE-luciferase reporter activity in S2R+ cells when treated with TONDU peptide. (D) Immunoblots showing competitive binding of TONDU peptide to Yki-Sd complex. IP, immunoprecipitation; TCL, total cell lysate. (E) Binding of TONDU peptide to Sd. (F-I) Guts from *esg^ts^>yki^3SA^* flies fed TONDU peptide: (F) unfed (control), (G) 50 μM (*n*=10), (H) 100 μM (*n*=12) and (I) 200 μM (*n*=10). (J) Quantification of GFP in TONDU peptide-fed and -unfed *esg^ts^>yki^3SA^* flies. Box plots indicate the median (horizontal lines), 25th and 75th percentiles (box), and 2.5 to 97.5 percentile range (whiskers). Outliers are displayed as filled circles. *P*-values for Student's *t*-test are displayed. (K) Viability of cancer cells on treatment with TONDU peptide, as estimated using the resazurin cell viability assay. Data presented as mean±s.e.; *P≤0.001 for Student's *t*-test. Scale bars: 10 µm (B), 100 µm (F-I).

Next, we asked whether feeding a synthetic TONDU peptide could inhibit Yki-driven ISC tumors comparable to its overexpression in ISCs (Fig. 1). We designed a synthetic peptide (Fig. 2A) derived from the TONDU domain of Vg that retained the conserved TEAD/Sd-interacting interfaces I and II containing the critical VXXHF motif (Pobbati et al., 2012). Further, since we aimed to administer the peptide orally to tumor-bearing flies, unlike in a previous study that involved tail vein injection of VGLL4-derived peptide (Jiao et al., 2014), we tagged the TONDU peptide with an HIV-TAT motif (RKKRRQRRR) and a nuclear localizing signal (NLS) (PKKKRKV) to facilitate cellular uptake (Wadia and Dowdy, 2005) and nuclear localization, respectively. Prior to oral administration of the peptide to adult flies, we first tested cellular uptake of a fluorescent-labeled TONDU peptide in S2R+ cultured cells, and observed its cytoplasmic and nuclear localization (Fig. 2B).

Further, to test whether the TONDU peptide can inhibit Yki-Sd complex formation, we used the Hippo response element (HRE)-luciferase reporter as a readout for Yki-Sd transcriptional activity (Wu et al., 2008). Specifically, we co-transfected S2R+ cells with the HRE-luciferase reporter along with Yki- and Sd-expressing vectors, treated the cells with 100 nM of TONDU peptide, and observed a moderate but consistent decrease in luciferase activity (Fig. 2C). Next, to confirm binding of the synthetic TONDU peptide to Sd and subsequent inhibition of Yki-Sd interaction, we carried out co-immunoprecipitation studies using S2R+ cells transfected with HA-Sd and GFP-Yki in the presence of the FLAG-tagged TONDU peptide. Indeed, we found that the TONDU peptide competitively inhibits binding of Sd to Yki (Fig. 2D). Finally, when purified HA-Sd from S2R+ cells was incubated with FLAG-tagged TONDU peptide, we observed binding with Sd, as revealed by immunoblots using anti-FLAG antibody (Fig. 2E). These observations are in agreement with previous studies (Guo et al., 2013; Koontz et al., 2013) that displayed binding of TONDU-containing protein Tgi to Sd via the TONDU domain. Together, these results indicate that TONDU peptide disrupts the Sd-Yki interaction by binding to Sd.

Next, we tested whether oral uptake of TONDU peptide inhibits *esg^ts^>yki^3SA^* ISC tumors. To first estimate the maximum tolerated dose, we examined the viability of *esg^ts^>GFP* flies when continuously fed different concentrations (25, 50, 100, 200 and 400 µM) of the TONDU peptide in standard fly food for 6-10 days at 29°C and scored their survival soon after. We observed that at 400 µM concentration of the TONDU peptide, only 55% (*n*=50) of the peptide-fed *esg^ts^>GFP* flies survived on day 6, whereas approximately 97%, 98%, 93% and 91% (*n*=50 in all cases) of flies survived at 25, 50, 100 and 200 µM of the peptide, respectively. We therefore fed *esg^ts^*>*yki^3SA^* flies, 24 h post-eclosion, 50, 100 or 200 µM TONDU peptide-supplemented food continuously for 10 days. Remarkably, we noted a progressive reduction in tumor load (Fig. 2F-I) with increasing concentration of TONDU peptide, as seen from a decrease in the numbers of GFP-marked ISCs (Fig. 2J). By contrast, tumor load was only moderately reduced when *esg^ts^*>*yki^3SA^* flies were fed food supplemented with sequence-scrambled TONDU peptide (Fig. S3A) at comparable concentrations (Fig. S3B-E); the residual inhibition of tumor growth observed with scrambled peptide could presumably be due to a partial retention of the secondary structure (Pobbati et al., 2012) of the TONDU peptide in the scrambled version as revealed by its predicted structure (Fig. S3F). We observed that, compared to poor survival (65.6% on day 10, Fig. 1G) of untreated *esg^ts^*>*yki^3SA^* flies, TONDU peptide-fed *esg^ts^*>*yki^3SA^* flies displayed a consistent increase in survival (68.2%, 74.3% and 79.7%) accompanying lowering of the tumor burden (Fig. 2G-I). We note, however, that peptide-fed flies continued to display some mortality, which we believe could be attributed to residual tumor load and/or off-target toxicity by the TONDU peptide *in vivo* due to perturbations in levels of a number of proteins in peptide-treated fly gut tumors (discussed below). Further, to confirm cellular uptake of TONDU peptide by the gut epithelia, we fed FLAG-tagged TONDU peptide (at a final concentration of 200 µM) to *esg^ts^*>*yki^3SA^* flies, and detected its cellular uptake in gut lysates by immunoblotting using an anti-FLAG antibody (Fig. S3G). In parallel, we also noted that feeding TONDU (at 200 µM) did not affect the numbers of ISCs in control guts (*esg^ts^>*GFP) (Fig. S3H).

In addition, we tested the tumor-inhibitory property of *Drosophila* TONDU peptide on human cancer cells. We observed that cell lines derived from human tumors with elevated *YAP1* levels (Fig. S3I), such as PC3 (prostate cancer), COLO-320 and WiDR (colorectal cancer), displayed growth arrest to varying extents upon uptake of TONDU peptide (Fig. 2K). On the other hand, the prostate cancer line LNCaP, which displayed negligible levels of *YAP1* (Fig. S3I), was not significantly inhibited by the TONDU peptide (Fig. 2K), even at higher concentrations, thereby revealing specificity of the TONDU peptide to inhibit YAP-mediated tumor growth and presumably low off-target toxicity. Altogether, these results suggest that TONDU is therapeutically relevant in YAP-driven tumors and can effectively inhibit cancers of different tissues of origin.

### Yki-driven tumor proteome reveals enrichment in integrin pathway components

**Fig. 3.**
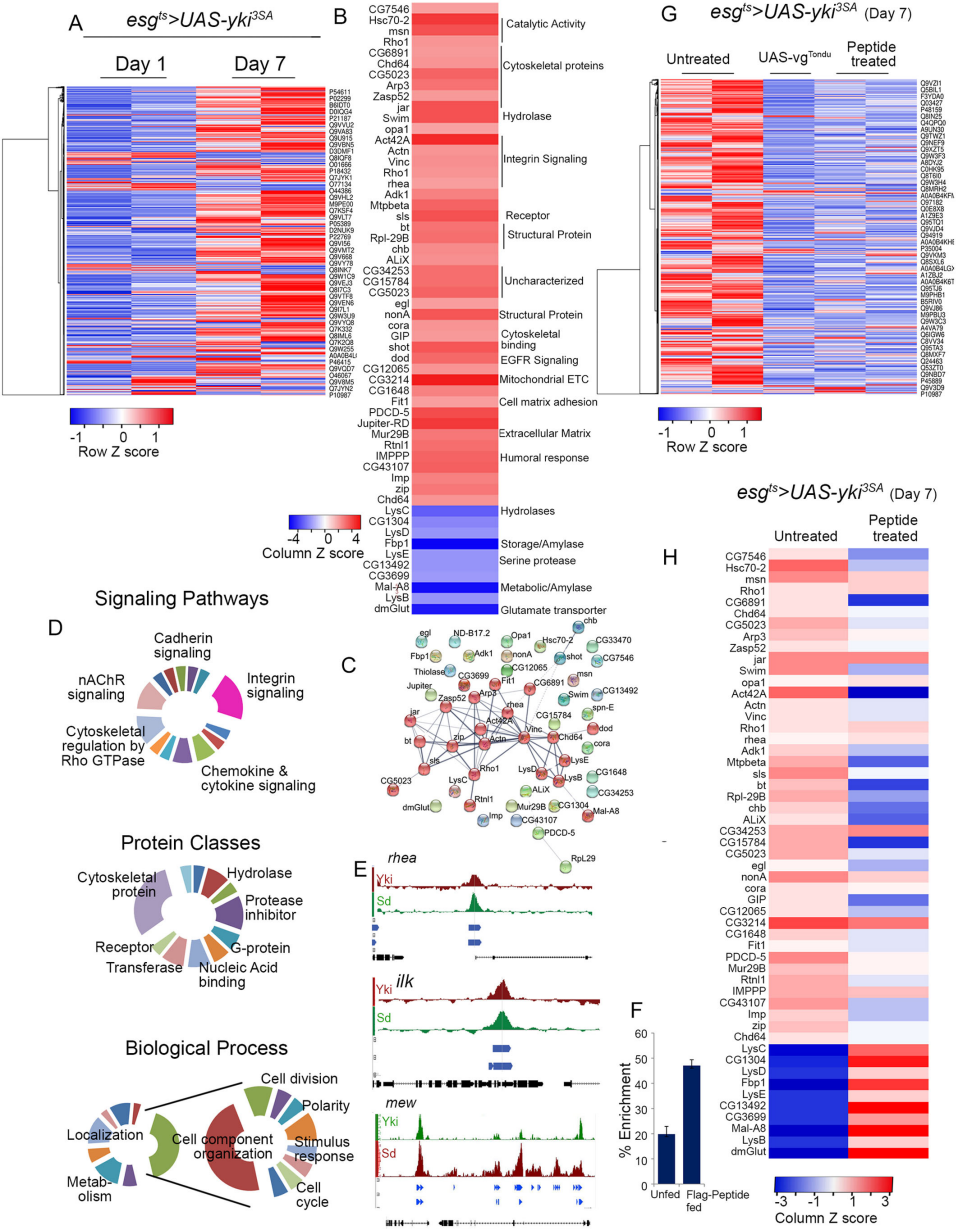
**Comparative proteomic analysis of Yki-driven ISC tumors and tumors inhibited by the TONDU peptide.** (A) Heat map displaying changes in protein levels in day 7 and day 1 *esg^ts^>yki^3SA^* tumors. (B) 55 differentially (>±2 log_2_fold, *P*=0.05) expressed proteins in day 7 *esg^ts^>yki^3SA^* tumors. (C) Protein-protein interaction (PPI) network of enriched proteins (>log_2_ 2 fold) in *esg^ts^>yki^3SA^* tumors generated with STRING (Szklarczyk et al., 2019), representing 55 nodes and 63 edges (PPI enrichment *P*<0.0001). (D) Different GO classes identified by PANTHER (Mi et al., 2007) in differentially expressed proteins between *esg^ts^>yki^3SA^* day 7 versus day 1 tumor proteome. (E) Sd and Yki binding sites in the regulatory regions of select integrin pathway members as determined in Nagaraj et al. (2012)*.* (F) Percentage enrichment for Sd binding upstream of *mew* (αPS1) inferred by ChIP with anti-FLAG antibody. (G) Heat map displaying the effect of TONDU peptide on *esg^ts^>yki^3SA^* tumor proteome. (H) Heat map displaying change in levels of protein (>±2 fold in day 7 tumors) upon TONDU peptide treatment.

We reasoned that significantly perturbed proteins in *esg^ts^>yki^3SA^* tumors, which are restored to normal levels following TONDU feeding, are likely to represent Yki-Sd targets that are crucial to ISC tumorigenesis, and, therefore, could be therapeutically relevant. Thus, we carried out a proteome analysis using unlabeled liquid chromatography–tandem mass spectrometry (LC-MS/MS) of *esg^ts^>yki^3SA^* tumors on day 1 and day 7 of tumor induction**,** with or without TONDU peptide-supplemented food. Altogether, we identified 1219 proteins (including isoforms), corresponding to 2771 unique UniProt IDs at a false discovery rate (FDR) cutoff of *q*<0.05 (Fig. 3A; Table S1) present in both day 1 and day 7 *esg^ts^>yki^3SA^* tumors. We next compared the proteomes of day 7 to day 1 *esg^ts^>yki^3SA^* tumors, and prioritized proteins that displayed at least a log_2_ ±2 fold change (at a *P*-value <0.05) for further analysis. Fold change was derived from the abundance measure of peptides (for a given protein) in day 7 versus day 1 *esg^ts^>yki^3SA^* tumors (see Materials and Methods). We identified 127 proteins (corresponding to 144 unique UniProt IDs, including isoforms) that were differentially expressed in day 7 *esg^ts^>yki^3SA^* tumors, and these matched to 55 unique genes (Fig. 3B; Table S2). Forty-five of these genes showed a greater than 2-fold (log_2_) increase and ten displayed a greater than 2-fold (log_2_) decrease in protein levels in day 7 compared to day 1 tumors (Table S2).

To further examine whether the proteins enriched in the ISC tumors are biologically relevant, we performed a protein-protein interaction (PPI) network analysis using STRING (Szklarczyk et al., 2019). We noted significant (*P*<0.001) interaction among some of the enriched proteins (Fig. 3C), suggesting that these were not random. Furthermore, we noted that the enriched gene set included known members of the Hippo protein-protein interaction network (Kwon et al., 2013), including junction proteins Coracle, Jar and Misshapen (Table S3). We also observed an increase in protein levels of the secreted Wg transporter Swim (Mulligan et al., 2012). Furthermore, comparison of the day 7 proteome of *esg^ts^>yki^3SA^* tumors with a recently published transcriptome of *esg^ts^>yki^3SA^* tumors (Song et al., 2019) revealed a close correlation between changes in proteins and their respective transcript levels (r=0.548) (Fig. S4A).

Signaling pathways perturbed in tumors are often causally linked to tumor progression (Khan et al., 2013; Bajpai and Sinha, 2020). We thus undertook a gene ontology (GO) classification of the proteins found enriched in ISC tumors to identify critical signaling pathways. GO classification using PANTHER (Mi et al., 2007) revealed perturbations in several signaling pathways and protein classes (Fig. 3D; Table S4). In particular, we observed an increase in protein levels of key members of the integrin signaling pathway, including Talin (2.39-fold; all fold changes have been mentioned at log_2_ conversion) and the Talin-interacting adaptor proteins Vinculin (2.4-fold) and Paxillin (6.05-fold). Other members, such as αPS3 and Integrin-linked kinase (Ilk), also displayed ∼2-fold change, albeit at *P*>0.05 (Table S5). Consistent with these findings, we noted transcriptional upregulation of the genes encoding the integrin members in the transcriptome of comparatively aged *esg^ts^>yki^3SA^* tumors (Table S5; also see Song et al., 2019), including *mew* (αPS1), *scb* (αPS3), *mys* (βPS), and integrin-binding ligands such as LanA and LanB (Table S5) (Song et al., 2019), which were otherwise not detected in the tumor proteome (Fig. 3B). It is likely that some integrin pathway components went undetected in proteomes owing to the limitation of unlabeled LC-MS/MS, such as failure to detect some proteins due to poor yield of their trypsinized products (Bantscheff et al., 2007). Significantly, we also observed an increase in levels of polarity proteins such as tight junction protein, Ferritin, Fit1 and the apical protein Shot (Fig. 3B), which are known to be regulated by the integrin pathway in the gut epithelium (Chen et al., 2018).

These observations suggest that genes encoding proteins enriched in *esg^ts^>yki^3SA^* tumors could be Yki-Sd transcriptional targets. To further examine this possibility, we searched for putative Yki-Sd binding sites from studies on genome-wide binding of Yki (Nagaraj et al., 2012; Oh et al., 2013) and Sd (Nagaraj et al., 2012). We noted that ∼51% (23 of 45) of the genes for which protein levels were increased in *esg^ts^>yki^3SA^* tumors displayed putative Sd and Yki binding sites in their upstream regulatory regions (Table S6). These included several key members of the integrin pathway, including *mew*, *vinculin*, *paxillin*, and *integrin-linked kinase* and *rhea* (Fig. 3E; Table S6). The integrin pathway has been reported to be essential for maintenance of both ISCs (Lin et al., 2013) and enterocytes (Chen et al., 2018); further, since αPS1 (encoded by *mew*) is particularly enriched in ISCs and critical for ISC maintenance (Lin et al., 2013), we sought to examine the role of the integrin αPS1 and the critical integrin-interacting protein Talin in *esg^ts^>yki^3SA^* tumors, despite αPS1 not being identified in the tumor proteome (Fig. 3B). Thus, we examined the binding of Sd to the upstream regulatory region of *mew*, and, given that TONDU peptide binds to Sd (Fig. 2D), we further reasoned that chromatin immunoprecipitation (ChIP) – using anti-FLAG antibody – on gut lysate of *esg^ts^>yki^3SA^* flies fed FLAG-tagged TONDU peptide could reveal binding of Sd. Indeed, we observed a significant enrichment of Sd binding in the upstream regulatory region of *mew*, *in vivo* in the guts of flies fed FLAG-tagged TONDU peptide (47.04±2.3%), compared to unfed control (19.8±3.0%) (Fig. 3F), suggesting that TONDU peptide binds to Sd and could therefore interfere with transcriptional regulation of *mew* by the Yki-Sd complex in *esg^ts^>yki^3SA^* tumors *in vivo*. By extension, it is likely that the TONDU peptide can also affect the expression of other transcriptional targets of Sd-Yki (Tables S2 and S6), including other members of the integrin pathway.

Next, to assess the impact of TONDU peptide on the tumor proteome, we compared the proteome of *esg^ts^>yki^3SA^* tumors with the proteome of *esg^ts^>yki^3SA^UAS-vg^TONDU^* and tumors from flies fed 200 µM of the peptide. Our earlier observation of comparable phenotypic suppression of *esg^ts^>yki^3SA^* tumors by either overexpression of TONDU peptide or its oral uptake, was supported by a strong correlation between their proteomes (Fig. S4B,C). Thus, we combined these two datasets for a robust representation of TONDU peptide-treated tumor proteome and compared it with that of untreated *esg^ts^>yki^3SA^* tumors. We observed an overall decrease in levels of proteins in peptide-treated ISC tumors compared to those of *esg^ts^>yki^3SA^* tumors from unfed controls (Fig. 3G). In addition, we noted that peptide-treated tumors displayed a significant decrease in protein levels (Fig. 3H), including the levels of critical members of the integrin pathway, such as Paxillin (−1.9-fold), Vinculin (−1.3-fold) and Talin (−1.2-fold) (Table S7).

Other notable perturbations included proteins involved in RNA processing, such as Pre-RNA processing factor 19 (Prp19) (−2.16-fold) (Guilgur et al., 2014) and Rumpelstiltskin (Rump) (−3.15-fold). Furthermore, decrease in Chromosome bows (Chb) (−2.16-fold), which is involved in mitotic spindle assembly (Reis et al., 2009), could presumably contribute to the lowering of cell proliferation of peptide-treated tumors. We also noted a decrease (−2.3-fold) in mitochondrial trifunctional protein β (Mtp-β), which catalyzes oxidation of long-chain fatty acids (Biswas et al., 2012), and could limit the energy source for peptide-treated tumors (Koundouros and Poulogiannis, 2020). Interestingly the tumor proteome revealed some novel and yet uncharacterized candidates that could be of functional significance. For instance, we noted a significant decrease in levels of proteins encoded by genes *CG15784* (−2.65-fold) and *CG7546* (−1.96-fold) in ISC tumors upon peptide treatment. Interestingly, these uncharacterized proteins interact with members of the Insulin/Akt (Vinayagam et al., 2016) and Hippo (Kwon et al., 2013) pathways, respectively, and could thus represent novel nodes connecting the Yki-oncogenic network with metabolic networks in the ISCs.

Taken together, our proteomic analyses reveal that TONDU peptide treatment perturbs a host of Yki-Sd targets that impinge upon cellular processes such as growth, proliferation and survival of tumor cells. It is likely that inhibition of *esg^ts^>yki^3SA^* tumors by the TONDU peptide could be a cumulative effect of suppression of multiple Yki-Sd targets affecting more than one signaling pathway or cellular process. We chose, however, to further examine the role of integrin signaling pathway since it plays a critical role in ISC maintenance (Lin et al., 2013).

### Genetic suppression of integrin signaling phenocopies TONDU-mediated suppression of Yki-driven ISC tumors

**Fig. 4.**
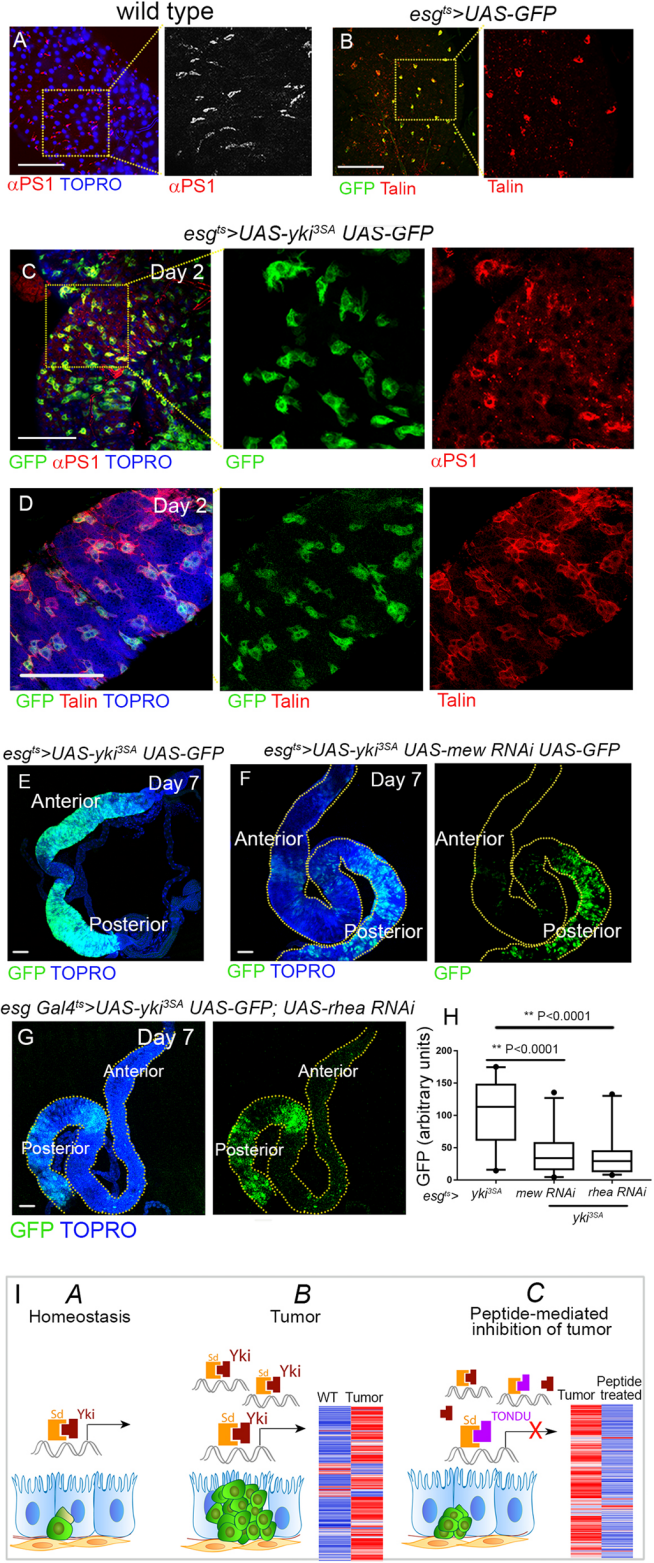
**Loss of integrin signaling inhibits growth of Yki-driven ISC tumors.** (A,B) αPS1 (A) and Talin (B) staining in *esg^ts^>UAS-GFP* marked ISCs. (C,D) Overall increase in αPS1 (C) and Talin (D) in *esg^ts^>yki^3SA^* tumors. (E-G) Inhibition of Yki-driven tumors upon simultaneous downregulation of αPS1 (*esg^ts^>yki^3SA^ UAS-mew-RNAi*, *n*=9; F) or Talin (*esg^ts^>yki^3SA^ UAS-rhea-RNAi*, *n*=9; G), when compared to similarly aged *esg^ts^>yki^3SA^* tumors (E and Fig. S5A, respectively). (H) Quantification of GFP from E, F and G. Box plots indicate the median (horizontal lines), 25th and 75th percentiles (box), and 2.5 to 97.5 percentile range (whiskers). Outliers are displayed as filled circles. Significance displayed as *P*-values, for unpaired Student's *t*-test. (I) Schematic of Yki-Sd mediated transcription in wild-type (WT) guts (A), in Yki-driven tumor (B) and in Yki-driven tumor in the presence of the TONDU peptide (C). Scale bars: 100 µm.

Integrins form an essential component of the *Drosophila* gut epithelia, including the basally located ISCs (Lin et al., 2013; Chen et al., 2018). Integrin, such as αPS1, is found specifically enriched in the ISCs (Lin et al., 2013) (also see Fig. 4A,B) and has been proposed to be important in the anchorage of the ISCs to the basement membrane and in their proliferation (Lin et al., 2013). Consistent with the enrichment of integrin pathway members in the *esg^ts^>yki^3SA^* proteome, we observed an overall increase in levels of integrin αPS1 (Fig. 4C) and Talin (Fig. 4D) in *esg^ts^>yki^3SA^* tumors. This observation, together with suppression of integrin pathway members such as Talin in TONDU peptide-fed flies suggests that integrin downregulation is likely to be causally linked to TONDU peptide-mediated inhibition of *esg^ts^>yki^3SA^* tumors. To test this possibility, we downregulated *rhea* or *mew* in the ISCs of *esg^ts^>yki^3SA^* tumors (Fig. 4E-G); indeed downregulation of *rhea* (*esg^ts^>yki^3SA^UAS-rhea-RNAi*, Fig. 4G) or *mew* (*esg^ts^>yki^3SA^UAS-mew-RNAi*, Fig. 4F) resulted in a marked reduction in ISC numbers (Fig. 4H), which was most obvious in the anterior midgut (Fig. 4F,G) when compared to similarly aged *esg^ts^>yki^3SA^* tumors (Fig. 4E; Fig. S5A). Moreover, examination of early (day 3) *esg^ts^>yki^3SA^UAS-mew-RNAi* guts revealed poor growth of ISC tumors. In particular, most of the ISC tumors were made up of small clusters of three to four cells (Fig. S5B,C), suggesting a strong decrease in tumor growth. These results are consistent with the observation that integrin signaling is required for ISC homeostasis (Lin et al., 2013). Interestingly, we note that gain of integrin alone, using a constitutively active form of the βPS integrin (Martin-Bermudo et al., 1999) in the ISCs (*esg^ts^>torso^D/βCyt^*) failed to trigger ISC proliferation (Fig. S5D). These observations suggest that although gain of integrin signaling alone per se does not transform ISCs, it is an essential partner for the progression of Yki-driven ISC tumors.

## DISCUSSION

TFs can be potent cancer drivers: suppression of TFs therefore constitutes a tumor inhibitory mechanism. The promise of targeting TFs for tumor therapy (Bhagwat and Vakoc, 2015; Lambert et al., 2018) is limited by the fact that small molecules often fail to target the large interacting surfaces associated with TFs (Lau and Dunn, 2018). Peptides, by contrast, have proven to be effective at interacting with large surfaces. Nevertheless, although peptides targeting extracellular receptors (Arosio et al., 2017) or intracellular inhibitors (Chang et al., 2013) have been explored, targeting of nuclear bound TFs with peptides remains poorly explored. The TEA/ATTS domain-containing TEAD proteins are a class of TFs that regulate YAP-induced proliferation and drive differentiation programs of VGLLs on the other (Gibault et al., 2018; Huh et al., 2019). In this study, using Yki-driven ISC tumors, we document *in vivo* inhibition of Yki-driven ISC tumor progression (Fig. 4I) by oral uptake of a *Drosophila* Vg-derived TONDU peptide. Remarkably, we observe a marked decrease in tumor load in TONDU peptide-fed flies, similar to what is observed upon ectopic expression of the peptide. Furthermore, a comparative proteomic analysis of ISC tumors in control and TONDU peptide-fed flies suggested a potential causal association between tumor suppression and downregulation of the integrin signaling pathway, a key player implicated in ISC homeostasis (Lin et al., 2013). Our study strongly suggests a critical role of integrin pathway in Yki-driven tumorigenesis; however, a mechanistic understanding of how these regulate each other in ISC tumorigenesis remains to be further elucidated.

TONDU peptide derived from VGLL4 was earlier shown to inhibit gastric tumors in mouse xenograft models (Jiao et al., 2014). Here, we further show the ability of TONDU peptide to inhibit proliferation of prostate and colon cancer cell lines with elevated YAP1 levels. It is likely that other solid tumors with activated YAP/TAZ (Zanconato et al., 2016) and/or TEAD proteins (Gibault et al., 2018; Huh et al., 2019) could be sensitive to inhibition by the TONDU peptide. Moreover, tumors that display loss of tumor suppressor VGLLs (Deng and Fang, 2018), and thereby activated TEAD protein, are likely targets of TONDU peptide-mediated inhibition. Further, our finding that inhibition of integrins suppresses Yki-driven tumors offers integrins as an alternative therapeutic target, which, being cell membrane localized, could be readily accessed (Ley et al., 2016). Finally, cross-species conservation of integrin signaling pathways (Cooper and Giancotti, 2019) and YAP/TAZ activity (Sebé-Pedrós et al., 2012) makes *Drosophila* tumor models ideal for exploring peptide and combinatorial therapeutic strategies for YAP-driven cancers.

It is noteworthy that peptide therapy in *Drosophila* has been successfully used to test therapeutic peptides that inhibit aggregate formation such as in Huntington's disease (Kazantsev et al., 2002) and Alzheimer's disease (Popiel et al., 2007), as well as peptides that exhibit immuno-modulatory roles (Pal et al., 2007). In most of these studies, however, peptides were injected into adult flies rather than administered orally. By contrast, oral administration of therapeutic peptides for treatment of human diseases, in general, carries the advantages of ease of administration, high patient compliance and, often, low production costs (Renukuntla et al., 2013). As is true for the development of small-molecule therapeutics, an *in vivo Drosophila* platform offers multiple advantages for peptide therapeutics, including scalability, genetic tractability and rapid elucidation of the mechanistic underpinning of peptide-based tumor suppression. *Drosophila* has emerged as a powerful model system to design and screen novel small-molecule drugs (Dar et al., 2012; Markstein et al., 2014; Bangi et al., 2016) as potential treatments for diverse diseases, including cancers. Our study expands the repertoire of *Drosophila* model-based screening options to include peptides.

Extrapolation of the TONDU peptide as a therapeutic for intestinal cancer is not without caveats. In mammals, intestinal cancers arise from multiple cell types: intestinal crypt stem cells (Barker et al., 2009), crypt progenitors or transit amplifying cells and, occasionally, via reprogramming of differentiated intestinal cells (Sadanandam et al., 2013). In a subset (Lgr5^+^) of crypt stem cells, gain of YAP surprisingly displays a tumor-inhibitory role via its cytoplasmic sequestration of disheveled 2 (DVL 2) (Barry et al., 2013) or by inhibiting the activity of the TCF transcriptional complex (Li et al., 2020). However, in intestinal crypt cells, activation of YAP drives their unrestricted proliferation (Camargo et al., 2007; Zhou et al., 2011; Li et al., 2020), resulting in intestinal adenomas; this pro-tumorigenic property of YAP is TEAD dependent (Li et al., 2020). Given the dual role of YAP – tumor suppression versus tumor promotion – in a cell type-specific manner, TONDU peptide-mediated therapeutic strategy may hold promise only in intestinal cancers that are mediated by the pro-tumorigenic YAP-TEAD complex. Indeed, a number of inhibitors targeting YAP/TAZ-TEAD complexes have now shown therapeutic promises in arresting growth of cancers, particularly those that display TEAD dependencies (for review, see Pobbati and Hong, 2020).

### Caveats and future directions

A major drawback of peptide therapeutics is the short half-life and poor bioavailability of the peptides. Use of non-natural amino acids (Verdurmen et al., 2011) and chemical modifications to stabilize the peptide backbone could help overcome these disadvantages (Furet et al., 2019). Moreover, oral administration of peptides presents additional challenges, including a need to survive harsh digestive milieu of the gastrointestinal tract and their enzymatic degradation (Renukuntla et al., 2013). Furthermore, the intestinal mucosa is found to act as a barrier to peptide absorption. Indeed addition of TAT domains that facilitate cellular uptake (Wang et al., 2017) to the TONDU peptide in our study could have contributed to the success of the oral TONDU peptide. Further improvement to stabilize therapeutic peptide to enhance bioavailability is a challenge for future work.

An additional challenge is that TFs can have multiple binding partners, such that targeting of a TF might result in off-target activities. For instance, with regard to the TONDU peptide, whereas *Drosophila* has a single TEAD protein, mammals have multiple TEAD proteins (TEAD1-4) that share the TONDU-interacting TEA/ATTS motif (Holden and Cunningham, 2018). This could lead to possible off-target activity of the TONDU peptide and consequent side effects. Further, since TEAD4 also binds to co-factors other than YAP and VGLLs such as the p160 nuclear receptors (Belandia and Parker, 2000), TONDU peptide administration might disrupt regulation by p160 of target genes, which include chromatic modifiers and epigenetic regulators. Identifying and limiting off-target activity of TONDU peptide therefore presents future goals essential for its therapeutic use.

## MATERIALS AND METHODS

### Fly lines, antibodies and primer sequences

Details on fly lines, antibodies and primer sequences used in the study are provided in Tables S8-S10.

### Genotypes of the flies used in the study

Genotypes of the flies used in the study are listed in Table S11.

### Induction of Yki-driven ISC tumors

We used the UAS-Gal4 system (Brand and Perrimon, 1993) to drive constitutively active Yki (*UAS-yki^S111A.S168A.S250A^*) in which three Serine phosphorylation sites are mutated (Oh and Irvine, 2009; Kwon et al., 2015), in the ISCs, using an ISC-specific Gal4 driver (*esg-Gal4*) under control of temperature-sensitive tub-Gal80^ts^ (Kwon et al., 2015). Flies were mated and maintained at 18°C until eclosion of the F1 generation. Freshly eclosed F1 flies of the genotype *esg>Gal4, tub-Gal80^ts^UAS-yki^3SA^* were shifted to 29°C and maintained until dissection.

### Generation of *UAS-vg^TONDU^* fly line

We synthesized an oligonucleotide coding for the *Drosophila* TONDU domain (CVVFTNYSGDTASQVDEHFSRALNY) (Pobbati and Hong, 2013). We introduced a start (ATG) and stop (TAA) codon flanking the nucleotide sequences, and inserted a 5′EcoR1 and 3′ Xba1 endonuclease restriction enzyme site on either side to allow directional cloning into pUASt vector (Addgene). We replaced Cytosine on position one and Alanine on position 22 with Serine (SVVFTNYSGDTASQVDEHFSRSLNY) to make the encoded peptide more polar and therefore improve its solubility. Substitution of terminal Cysteine would also reduce chances of aberrant dimer formation. The VXXHF domain of the TONDU domain, which is essential for interaction with TEAD/Sd (Pobbati et al., 2012), was left unchanged. The synthesized oligonucleotide was cloned into pUAST vector carrying *mini white*, and injected into Canton S embryos at the Center for Cellular and Molecular Platforms (C-CAMP), National Centre for Biological Sciences (NCBS), Bangalore, India. Adults were screened for insertion of the vector into the third chromosome.

### Design and synthesis of the TONDU peptide and its variants

#### TONDU peptide

We synthesized a peptide corresponding to the TONDU domain with certain modifications. The basic peptide is a 46-amino-acid peptide (YGRKKRRQRRRGGPKKKRKVGG [VVFTNYSGDTASQVDEHFSRALNY]) comprised of 24 amino acids of the TONDU domain (VVFTNYSGDTASQVDEHFSRALNY) preceded by the conserved SV40 T-antigen nuclear localizing signal (PKKKRKV) (Lanford et al., 1986) and a cell-penetrating peptide (YGRKKRRQRRR) derived from human immunodeficiency virus (HIV) (Vivès et al., 1997); the NLS sequence was flanked by a di-glycine (GG) spacer to avoid any steric hindrance between the tag and the rest of the peptide. The first Cytosine on the TONDU domain was removed to prevent dimerization of the peptide.

#### FLAG-tagged TONDU peptide

To test for binding partners to TONDU peptide, we added a FLAG tag (DYKDDDDK) at its C-terminus (YGRKKRRQRRRGGPKKKRKVGG-VVFTNYSGDTASQVDEHFSRALNYDYKDDDDK) to allow protein immunoprecipitation using an anti-FLAG antibody.

#### Fluorescent-tagged TONDU peptide

To track uptake of the peptide and facilitate its cellular localization, we added 5-TAMARA, a fluorescent tag, to the C-terminus of the TAT-NLS-TONDU peptide. The peptides were synthesized at GL Biochem (Shanghai, China).

#### Administration of TONDU peptide

Lyophylized TONDU peptide was dissolved in water to a final concentration of 1 mM (used as stock), which was then used to prepare 50, 100, 200 or 400 µM of working stock. Then, 100 µl of each was sprayed over freshly cooled standard fly food (not containing any anti-fungal or anti-bacterial agent), on which flies were reared. The flies were transferred into fresh vials (containing TONDU peptide) every 24 h for 10 days.

### Immunostaining of *Drosophila* adult midguts

Prior to dissection, female flies of desired genotype were starved briefly and fed water for 2 h to flush out food from the gut. Midguts were dissected in 1× PBS and fixed in 4% paraformaldehyde in PBS containing 0.2% Triton X-100 for 30 min at room temperature, followed by washing in PBS containing 0.2% Triton X-100 for 15 min. The guts were then incubated in primary antibody at 4°C overnight, followed by blocking with 0.1% bovine serum albumin (BSA) for 1 h and incubation with secondary antibody (Alexa Fluor 555 against mouse or rabbit) for 4 h at room temperature. Next, guts were washed in 1× PBS and counterstained for nuclei using TO-PRO-3 (Invitrogen, S33025) or F-actin using Alexa Fluor Phalloidin-633 (Invitrogen, A22284; 1:100), followed by mounting in an anti-fade mounting medium, Vectashield (Sigma-Aldrich).

### Microscopy and image processing

Images were acquired using a Leica SP5 confocal microscope and processed using the Leica application software and Adobe Photoshop CS5.

### Measurement of GFP from confocal images

GFP was quantitated from full projections of images acquired using confocal microscopy. GFP intensity in gray scale from regions of interest (ROIs) covering the entire gut was acquired using the Leica-LSM proprietary software. GFP intensity was normalized to the area of each ROI. Student’s *t*-test was performed using MS Excel to look for statistical significance in GFP variation.

### EdU cell proliferation assay

Cell proliferation was detected by 5-ethynyl-2 deoxyuridine (EdU) uptake using a Click-iT Alexa-Fluor-555 290 kit by Invitrogen. Briefly, unfixed guts from female *esg^ts^>UAS-yki^3SA^* flies were incubated with 100 μM EdU in Schneider's insect medium, for 1 h at room temperature. Tissue was then fixed in 4% paraformaldehyde and incubated in secondary buffer containing fluorescent-tagged dye (following the manufacturer's instructions) for 1 h at room temperature and subsequently washed in PBS, counter-stained with TO-PRO-3 (Invitrogen, S33025) and mounted using an anti-fade mounting medium (Invitrogen).

### Quantitative RT-PCR

Real-time PCR (RT-PCR) was performed using SYBR green (Applied Biosystems) on ABI7 900 HT. Prior to dissection, *esg^ts^>UAS-yki^3SA^* females were starved briefly and fed water for 2 h to flush out food from the gut. Total RNA from 20 midguts was isolated using Qiagen RNeasy columns. For human cancer cells, total RNA was extracted using TRIzol (Ambion). RNA was treated with RNase-free DNase (Roche) to get rid of any traces of DNA before converting RNA to complementary DNA (cDNA) using a cDNA preparation kit (Invitrogen). The resulting cDNA was used as substrate for relative quantitation using SYBR green on ABI7 900 HT. β-Tubulin was used as an endogenous control. Genes were assayed from four biological replicates for each condition. Quantitative PCR (qPCR) was performed using the following conditions: DNA polymerase activation for 10 min at 95°C, followed by 40 cycles of duplex melting for 15 s at 95°C and a combined annealing and extension step for 1 min at 60°C. The threshold-cycle (Ct) values were generated automatically. The relative expression value of each gene in the two conditions was calculated using the 2−ΔΔCt method.

### Cancer cell line and cell culture conditions

The prostate (PC3 and LNCaP) and colorectal (COLO 320-HSR) cancer cell lines were obtained from American Type Cell Culture (ATCC; Manassas, VA, USA). The colorectal cancer cell line WiDr was a kind gift from Dr Eric R. Fearon, University of Michigan, Ann Arbor, MI, USA. All of the cell lines were cultured as per ATCC guidelines in a CO_2_ incubator (Thermo Fisher Scientific) supplied with 5% CO_2_ at 37°C. Cell line authentication was done via short tandem repeats (STR) profiling at Lifecode Technologies Private Limited (Bangalore, India) and DNA Forensics Laboratory (New Delhi, India). Routine check for mycoplasma contamination of all cell lines was carried out using a PlasmoTest mycoplasma detection kit (InvivoGen).

### Cell viability assay of human cancer cell lines

To determine the effect of TONDU peptides on the cell viability of prostate cancer (PC3 and LNCaP) and colorectal cancer (COLO320 and WiDR) cells, ∼3000 cells were plated in each well of a 96-well plate. After 24 h, TONDU peptide was added to the cultured cells at three different concentrations: 50 nM, 100 nM and 250 nM. No peptide was added in the control group. After 72 h and 96 h of peptide treatment, cell viability was determined using resazurin sodium salt solution (R7107, Sigma-Aldrich). Briefly, resazurin (0.02 mg/ml; w/v) diluted in culture medium was added to the cells and incubated for 4 h in the dark at 37°C. Fluorescence was measured at 530/590 nm (excitation/emission) using a Synergy™ H4 Hybrid Microplate Reader (BioTek, Winooski, VT, USA). Three biologically independent samples were used in each experiment; data represent mean±s.e.m. Statistical significance was determined using two-tailed unpaired Student's *t*-test.

### Immunoprecipitation studies to determine binding of TONDU peptide to Sd

*Drosophila* S2R+ cells (sex: male) were cultured in Schneider's medium supplemented with 10% fetal bovine serum (FBS) at 25°C. Full-length Sd (GEO03367) and Yki (GEO02945) cDNAs from the *Drosophila* Genomics Resource Center were cloned into the *Drosophila* Gateway vector pAWH and pAWG, respectively. GFP was cloned into pAWM as a control.

Immunoprecipitation and immunoblotting were performed as previously described (Tang et al., 2018). In brief, DNA was transfected into S2R+ using Effectene transfection reagent (Qiagen, 301427). After 2 days of incubation, cells were incubated with or without 1 µM TONDU peptide for 24 h and then lysed with lysis buffer (Pierce, 87788) containing a protease and phosphatase inhibitor cocktail (Pierce, 78440). Lysate was incubated with Chromotek-GFP-Trap (Bulldog Biotechnology, gta-20) for 2 h at 4°C to precipitate the proteins. Beads were washed three to four times with 1 ml lysis buffer and then boiled in SDS sample buffer, run on a 4-20% polyacrylamide gel (Bio-Rad, 4561096), and transferred to an Immobilon-P polyvinylidene fluoride (PVDF) membrane (Millipore). The membrane was blocked by 5% BSA in TBST (Tris-buffered saline with 0.1% Tween 20) at room temperature for 1 h and then probed with anti-GFP (Molecular Probes, A6455), anti-hemagglutinin (HA) (Covance/BioLegend, MMS-101P) or anti-FLAG (Sigma-Aldrich, F3165) antibody in 1× TBST with 5% BSA overnight, followed by horseradish peroxidase (HRP)-conjugated secondary antibody, and signal was detected by enhanced chemiluminescence (Amersham, RPN2209; Pierce, 34095).

For the TONDU-Sd binding assay, HA-Sd was expressed in S2R+ cells and purified through immunoprecipitation with RIPA buffer (Pierce, 89901) and anti-HA agarose (Sigma-Aldrich, A2095). Purified HA-Sd proteins were incubated with 1 µM TONDU peptide directly. The sample was then washed and subjected to immunoblotting.

### Quantitation of the effect of the TONDU peptide on Yki-Sd-driven transcription using HRE

#### Luciferase reporter

*Drosophila* S2R+ cells were maintained at 25°C in Schneider's medium (GIBCO) with 10% heat-inactivated FBS (Sigma-Aldrich) and 5% Pen-Strep (GIBCO). Experiments were run on 24-well plates, with three replicates per condition. Cells were co-transfected with 100 ng each of HRE-luciferase reporter [containing two copies of an HRE cloned upstream of an hsp70 basal promoter in pGL3 basic vector (Wu et al., 2008)], along with Sd- or Yki-expressing pAc5.1/V5-HisB plasmids (Wu et al., 2008) (gift from Duojia Pan, UT Southwestern Medical Center, Dallas, TX, USA); 10 ng Act-Renilla was used for transfection control. Transfection was carried out using Effectene (Qiagen), as per the manufacturer's recommended protocol. Then, 24 h after transfection, 50 nM or 100 nM of the TONDU peptide was added to wells in triplicate, and 48 h after addition of the TONDU peptide, cells were harvested and luciferase activity was measured using Dual Glo (Promega) as per the kit instructions, measured using a Spectramax Luminescence plate reader.

#### Detection of fluorescent-labeled TONDU peptide in S2R+ cells

*Drosophila* S2R+ cells were grown to confluence in Schneider's medium (GIBCO) supplemented with 10% heat-inactivated FBS (Sigma-Aldrich) and 5% Pen-Strep (GIBCO) at 25°C in 24-well plates. TAMARA-tagged TONDU peptide was added to the medium to a final concentration of 100 nM and cells were incubated for 6 h. Next, the medium was discarded and cells were washed three times with 1× PBS. Cells were then added to lysine-coated slides, fixed with 4% formaldehyde in 1× PBS and counterstained with 4′,6-diamidino-2-phenylindole (DAPI). Cells were imaged with a Nikon Ti, CSU-X1 spinning disk confocal microscope and the images were processed using Fiji image processing software (https://imagej.net/Fiji).

#### ChIP to determine binding of TONDU peptide to Sd in the upstream regulatory region of gene *mew*

ChIP was performed using LowCell# ChIP kit protein A (Diagenode, C01010072) according to the manufacturer's instructions. Briefly, midguts from 35 adult *esg^ts^>UAS-yki^3SA^* females (pre-starved for 1 h) were dissected in ice-cold 1× PBS and crosslinked in 1% formaldehyde (Sigma-Aldrich) for 15 min at 37°C. Crosslinking was quenched with 125 mM glycine. The guts were washed with PBS and precipitated by centrifugation at 3500 ***g*** for 5 min. The pellet was lysed in 250 ml Buffer B (LowCell# ChIP kit) supplemented with complete protease inhibitor (Roche) and phenylmethylsulfonyl fluoride (PMSF; Sigma-Aldrich). Lysed chromatin (130 µl) was sheared using a Bioruptor (Diagenode) at high frequency for 15 cycles of 30 s ON, 30 s OFF. Then, 870 µl of Buffer A (LowCell# ChIP kit) supplemented with complete protease inhibitor (Roche) and PMSF (Sigma-Aldrich) was added to the shared chromatin, and 8 µl of the chromatin solution was saved as an input control. Magnetic beads (11 µl) were washed twice with Buffer A (LowCell# ChIP kit) and resuspended in 800 µl Buffer A. Anti-FLAG antibody (2 µg; Sigma-Aldrich, F1804) was then added to the washed beads and gently agitated at 4°C for 4 h. The beads-antibody complex was precipitated with a magnet and the supernatant was removed, and 800 µl shared chromatin was added to the beads-antibody complex and rotated at 4°C overnight. The immobilized chromatin was then washed with Buffer A three times and Buffer C once, and eluted in 100 µl elution buffer (1% SDS, 0.1 M sodium bicarbonate with proteinase K and RNaseA). The chromatin was subjected to either phenol-chloroform extraction for DNA purification and subsequent qPCR analysis, or the protein was extracted by heating the washed beads at 95°C in 20 μl SDS loading dye (4×) for 10 min and centrifuged at 15,000 ***g*** for 10 min. The supernatant was collected and used for dot blot analysis.

#### Protein dot blot

TONDU peptide (1 mM) was serially diluted (10^−1^, 10^−2^, 10^−3^) and blotted using a narrow-mouth pipette tip, and 7.5 µl peptide or enriched protein fraction from ChIP was applied slowly onto the nitrocellulose membrane (Thermo Fisher Scientific, 0.2 μm pore size). The membrane was air dried and then blocked in 5% BSA in TBST for 2 h at room temperature, then incubated for 3 h with a secondary antibody conjugated to HRP (Jackson ImmunoResearch, 711035152), washed three times with TBST, detected with chemiluminescent substrate (Thermo Fisher Scientific, 34080) and visualized on X-ray film (Fuji, Super HR-t).

### Proteomics of Yki-driven ISC tumors

#### Protein extraction from fly guts for LC-MS/MS analysis

Prior to dissection, female *esg^ts^>UAS-yki^3SA^* flies were briefly starved and fed water for 2 h to clear the gut. Adult guts were dissected in cold 1× PBS from 20 flies. The fore- and hindguts were removed, and the midguts were put in 100 µl extraction buffer (6 M GnHCl in 50 mM Tris-HCl pH 7.4, 65 mM dithiothreitol) with 50 mM sodium acetate and protease inhibitors (1× protease inhibitor cocktail with 0.2 mM PMSF) was added to the sample. The guts were sonicated with a Bioruptor (Diagenode) using the following settings: sonication cycle: 30 s ON and 30 s OFF for 5 cycles at 4°C. Cell debris was removed by centrifuging at 6000 ***g*** for 3 min; then the supernatant was transferred to a new tube. The protein concentration was determined spectrophotometrically using Nanodrop and BCA protein assay (Thermo Fisher Scientific), following the manufacturer's protocol. Five micrograms of the protein were used for LC-MS-MS analysis, and we made certain that the tissue was processed within 30 min of dissection.

Sample preparation for LC-MS/MS

Five micrograms of the protein samples were reduced with 5 mM tris(2-carboxyethyl)phosphine (TCEP), further alkylated with 50 mM iodoacetamide and digested with Trypsin (1:50, Trypsin/lysate ratio) for 16 h at 37°C. Digests were cleaned using a C18 silica cartridge to remove the salt and dried using a speed vac. The dried pellet was resuspended in 5% acetonitrile, 0.1% formic acid (Buffer A).

Mass spectrometric analysis of peptide mixtures

The experiment was performed using an EASY-nLC 1000 system (Thermo Fisher Scientific) coupled to a Thermo Fisher-Orbitrap Fusion mass spectrometer equipped with a nanoelectrospray ion source. One microgram of the peptide mixture was resolved using a 25 cm Thermo Easy-spray PepMap C18 column. The peptides were loaded with Buffer A and eluted with a 0-40% gradient of Buffer B (95% acetonitrile, 0.1% formic acid) at a flow rate of 300 nl/min for 60 min. Mass spectrometry (MS) data were acquired using a data-dependent top 20 method, dynamically choosing the most abundant precursor ions from the survey scan. The LC-MS/MS RAW files have been submitted to MassIVE repository (https://massive.ucsd.edu) and can be accessed using MSV000084841.

#### Data processing

All samples were processed and the eight RAW files generated were analyzed with Proteome Discoverer (v2.2) against the UniProt *Drosophila melanogaster* reference proteome database. For Sequest search, the precursor and fragment mass tolerances were set at 10 ppm and 0.5 Da, respectively. The protease used to generate peptides, i.e. enzyme specificity, was set for trypsin/P (cleavage at the C terminus of ‘K/R: unless followed by P’) along with a maximum missed cleavages value of two. Carbamidomethyl on cysteine as fixed modification and oxidation of methionine and N-terminal acetylation were considered as variable modifications for database search. Both the peptide spectrum match and the protein FDR were set to 0.01 FDR.

#### Proteome data analysis

To identify biologically relevant protein signatures in *esg^ts^>yki^3SA^* tumors and characterize their status in the presence of the TONDU peptide, we calculated the log2 abundance ratios, using mean abundance values for individual UniProt IDs of *esg^ts^>UAS-yki^3SA^* day 7 versus day 1 proteome. Only those with combined FDR confidence <0.05 (medium) or <0.01 (high) were taken into consideration; those with combined FDR >0.05 (low) were discarded. We further filtered out peptides that were not detected in either MS or MS/MS spectra, depending on the peak calling. We noted that the number of peptides that matched each UniProt ID, ranged from 1 to 67. To ascertain statistically significant calls, we applied Student’s *t*-test on replicate readings for the individual UniProt IDs and only those with *P*<0.05 were considered. We first calculated log_2_ abundance ratio of proteins in day 7 with day 1 of *esg^ts^>yki^3SA^* tumors, and considered only gene products for which log2 fold change was ≥2. Next, we examined the status of tumor proteins from TONDU peptide-fed flies. Since the TONDU-peptide treated tumors phenocopied the tumor suppression seen by overexpression of the TONDU peptide (compare Fig. 1 with Fig. 2), and their protein profiles displayed closed correlation (Fig. S4), we chose to combine these two data sets. We therefore calculated log_2_ abundance ratio of individual proteins in untreated day 7 *esg^ts^>yki^3SA^* tumors, to that of TONDU peptide-treated and to TONDU-peptide expressed (*UAS-vg^TONDU^*) ISC tumors. We applied the Student's *t*-test to look for statistical significance for each log2 fold change and considered only those with *P*<0.05.

#### GO analysis

To identify the biological function of genes and look for enrichment of functional classes, we undertook GO analysis using the Protein ANalysisTHroughEvolutionaryRelationships (PANTHER) classification system (http://www.pantherdb.org; Mi et al., 2007). Protein functions were inferred by classification of genes into one or more groups, depending on: (1) molecular function, (2) biological process, (3) protein class, (4) pathways and (5) cellular component.

#### Heat maps

Heat maps were generated using Heatmapper (http://heatmapper.ca/). For the heat map in Fig. 3A, raw abundance values for individual UniProt IDs were subjected to row scaling, and clustered using average linkage clustering with Euclidean method for distance measure.

## Supplementary Material

Supplementary information
