# Supplementary material for: A Drosophila model of oral peptide therapeutics for adult intestinal stem cell tumors
Source: Dis Model Mech. 2020 Jul 24;13(7):dmm044420. doi: 10.1242/dmm.044420 (PMC7390633; doi:10.1242/dmm.044420)
Supplement: Supplementary information [file dmm-13-044420-s1.pdf]

FIGURE S1

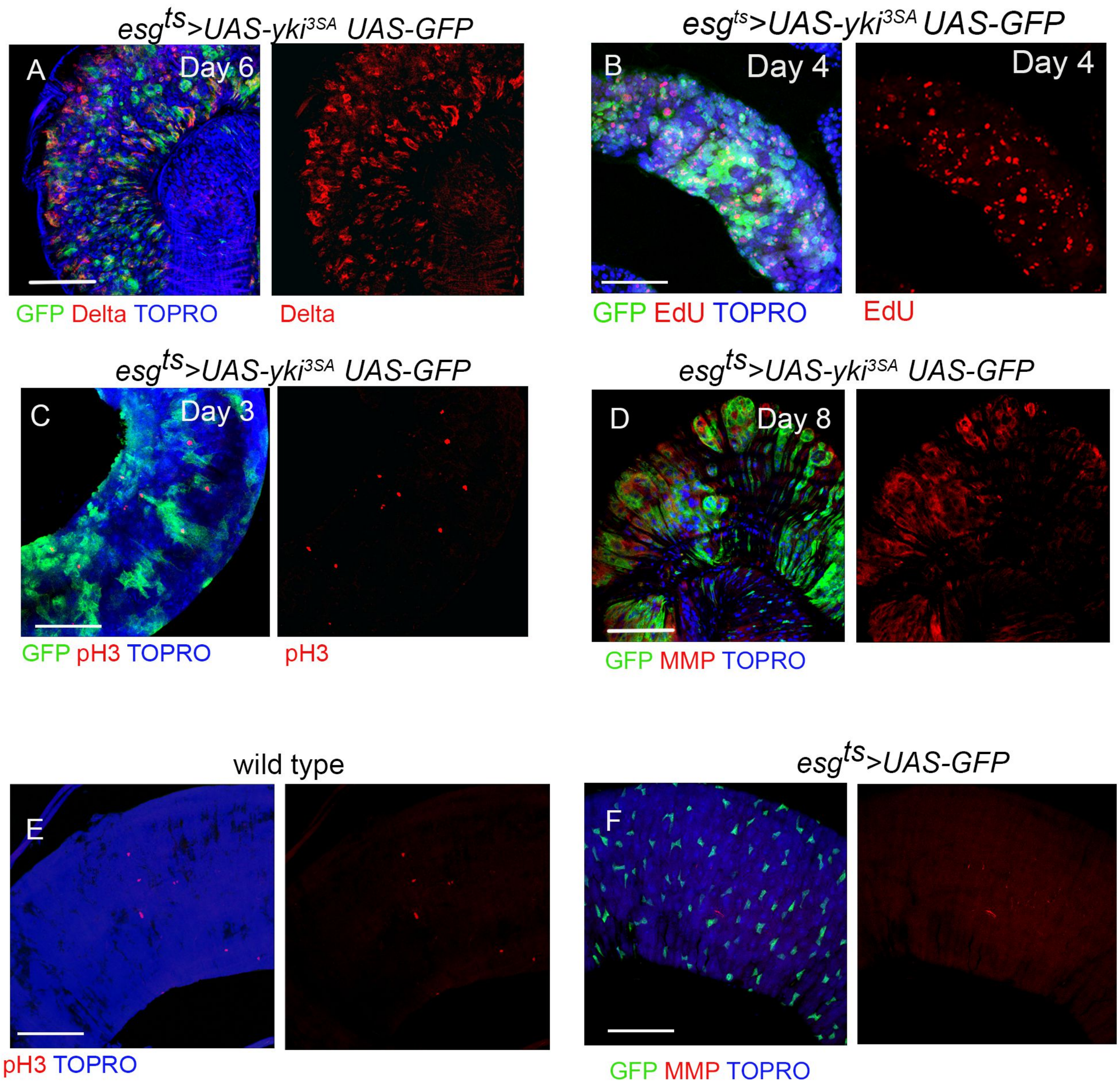

Figure S1. ISCs with constitutive gain of Yki display tumor phenotypes. (A-D) *esg*^{ts}*>yki*^{3SA} *UAS-GFP* tumors stained for the stem cell marker Delta (A), EdU uptake (B), Phospho-Histone (C), and MMP (D). (E-F) Wild-type guts displaying Phospho-Histone (E), and MMP (F). Scale bars 100 μm.

Disease Models & Mechanisms • Supplementary information

FIGURE S2

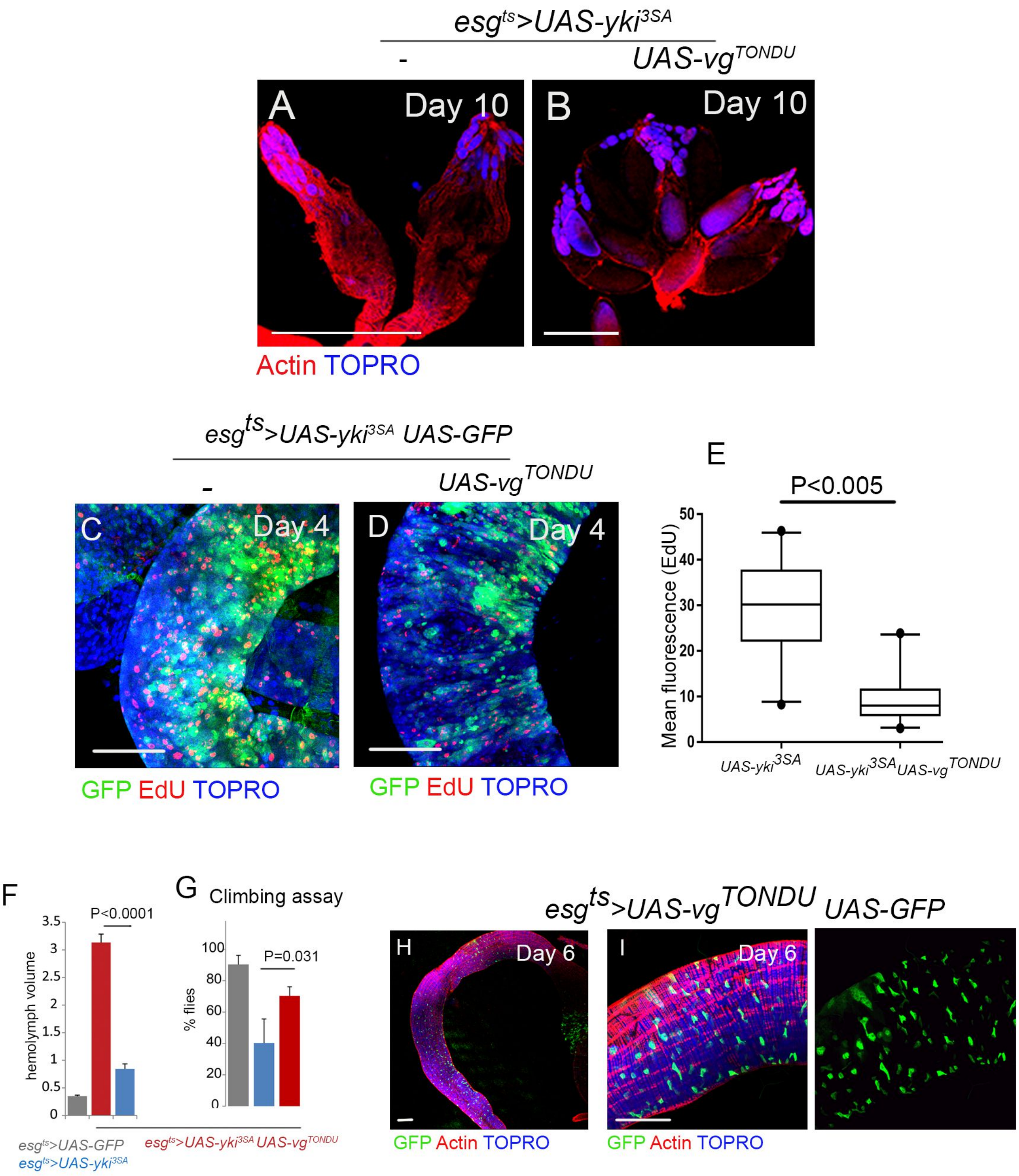

Disease Models & Mechanisms • Supplementary information

Figure S2. Overexpression of the TONDU peptide inhibits Yki-driven epithelial tumors. (A) Atrophy of ovaries in *esg*^{ts}*>yki*^{3SA} flies (n=21/25). (B) Improved morphology of ovaries in *esg*^{ts}*>yki*^{3SA} *UAS-vg*^{TONDU} flies (n=12/25). (C-E) Decrease in the number of proliferating cells detected by EdU (red) staining in *esg*^{ts}*>yki*^{3SA} *UAS-vg*^{TONDU} (D), compared to *esg*^{ts}*>yki*^{3SA} tumors (C). (E) Quantification of EdU fluorescence in C and D. Box plots indicate the median (horizontal lines), 25th and 75th percentiles (box) and 2.5 to 97.5 percentile range (whiskers). Outliers are displayed as filled circles. P values for Students t-test are displayed. (F) Decrease in hemolymph content (n=25) in *esg*^{ts}*>yki*^{3SA} *UAS-vg*^{TONDU} flies compared to *esg*^{ts}*>yki*^{3SA} flies on Day 7. (G) TONDU-expressing *esg*^{ts}*>yki*^{3SA} flies (n=35) suppress the loss of climbing activity seen in *esg*^{ts}*>yki*^{3SA} flies. (H-I) Expression of TONDU peptide in ISCs (*esg*^{ts}*>UAS-vg*^{TONDU}) does not affect ISC numbers. Scale bars 100 µm.

Disease Models & Mechanisms • Supplementary information

Figure S3

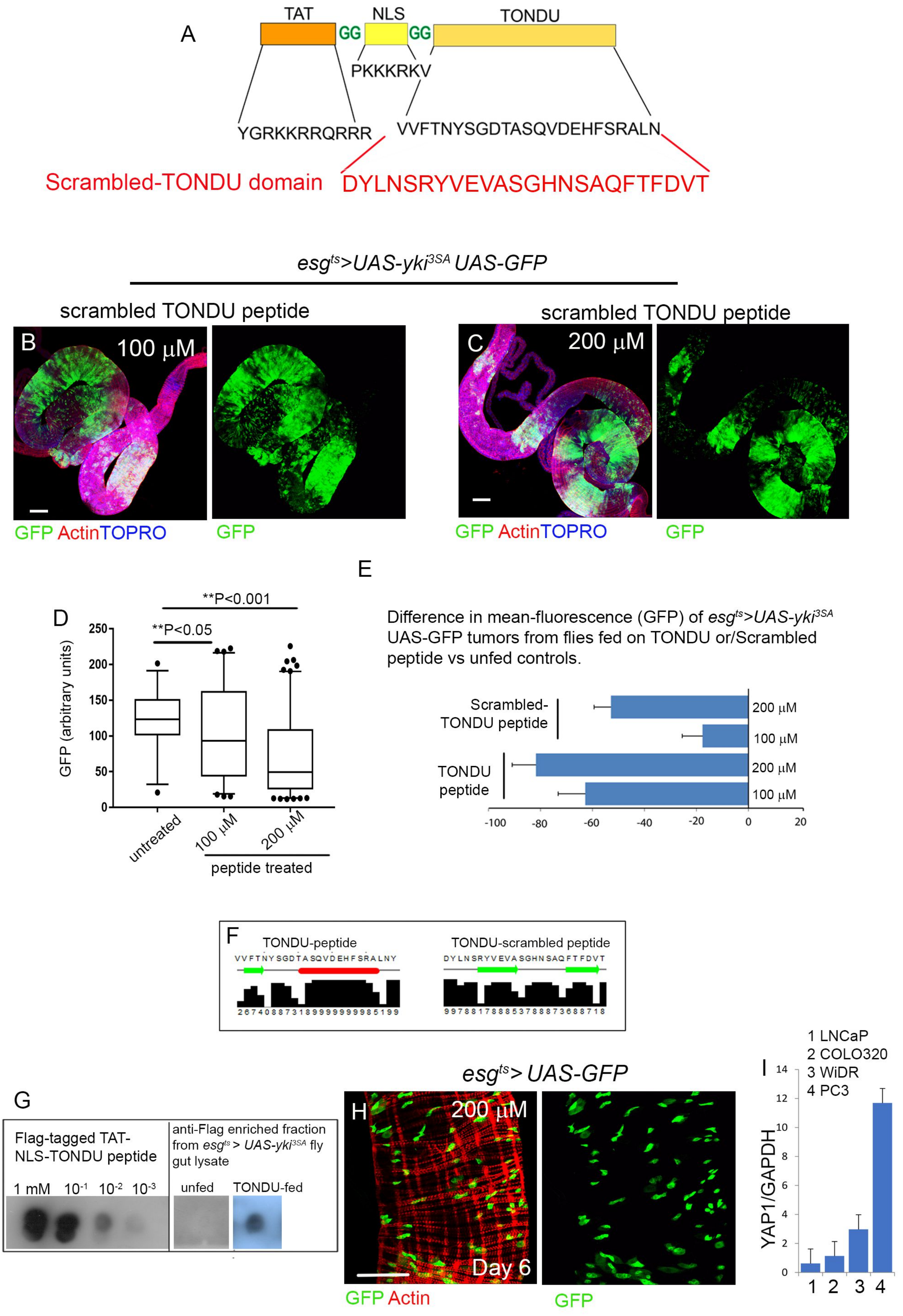

Disease Models & Mechanisms • Supplementary information

Figure S3. Scrambled-TONDU peptide fails to suppress Yki-driven ISC tumors. (A) Schematic representation of the scrambled-TONDU peptide. (B-D) The scrambled-TONDU peptide displays poor growth inhibition of $esg^{ts}>yki^{3SA}$ tumors (compare with Figure 2H and I). Box plot depicting GFP quantification in $esg^{ts}>yki^{3SA}$ tumors from flies fed on scrambled TONDU peptide (D). Box plots indicate the median (horizontal lines), 25th and 75th percentiles (box) and 2.5 to 97.5 percentile range (whiskers). Outliers are displayed as filled circles. P values for Students t-test are displayed. (E) Histogram displaying decrease in mean-GFP of $esg^{ts}>yki^{3SA}$ *UAS-GFP* tumors from flies fed with TONDU peptide or with scrambled-TONDU peptide, when compared to unfed controls. Note that the decrease is significantly more in TONDU peptide as compared to scrambled peptide fed tumors. (F) Secondary structures of the TONDU (left) and Scrambled-TONDU (right) as predicted by JPred (http://www.compbio.dundee.ac.uk/jpred/). Black bars represent the confidence estimate for the prediction (high value represents high confidence); helices are marked in red while green arrows depict sheets. (G) Dot blot for FLAG-tagged TONDU peptide using anti-FLAG antibody, on native peptide (different serial dilutions); and in cell lysate (right panel) from guts (n=25) of flies fed on 200 µM of FLAG-tagged TONDU peptide and unfed flies used as control. (H) Control ($esg^{ts}>UAS\text{-}GFP$) flies fed on 200 µM of TONDU peptide do not display changes in ISC numbers. (I) mRNA levels of *YAP1* in different human cancer cell lines as determined by qPCR. Scale bars 100 µm.

Disease Models & Mechanisms • Supplementary information

Figure S4

X-Y plot depicting correlation between proteome and transcripome of *esg^{ts}>yki^{3SA}* tumor

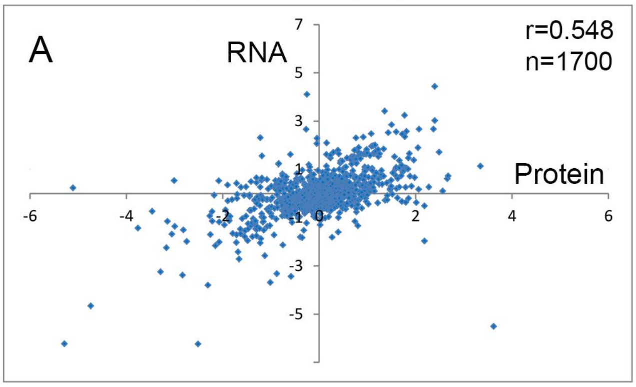

Scatter plot displaying fold change in individual proteins in *esg^{ts}>yki^{3SA}* tumors in presence of TONDU peptide

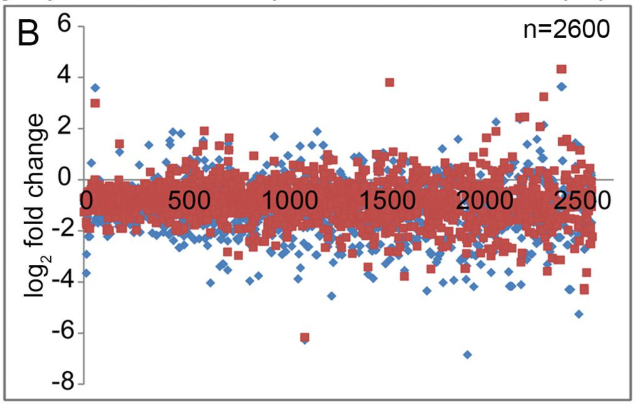

x axis: individual proteins
y axis: fold change

■ *esg^{ts} >yki^{3SA}* tumors from flies fed on TONDU peptide *versus* unfed controls.

■ *esg^{ts}>yki^{3SA}; UAS-vg^{TONDU} versus esg^{ts}>yki^{3SA}* tumors

X-Y plot depicting correlation between TONDU peptide-treated and TONDU peptide-expressed *esg^{ts}>yki^{3SA}* tumor proteome

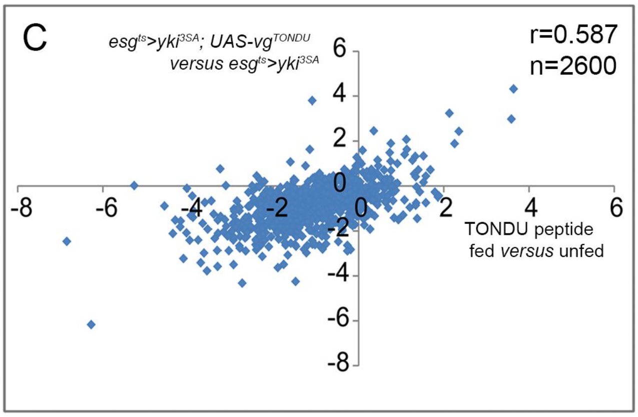

Figure S4. Comparison of the proteomes of TONDU peptide-treated ISC tumors and tumors with genetic gain of peptide. (A) X-Y correlation plot displaying Z-score comparison of $\log_2$ fold change of genes in the proteome (current study) and transcriptome (Song et al., 2019) of *esg*^{ts}>*yki*^{3SA} tumors. (B) Scatter plots displaying fold change ($\log_2$) for individual proteins (x axis) in TONDU peptide-fed versus unfed e*sg*^{ts}>*yki*^{3SA} tumors (red); and for *esg*^{ts}>*yki*^{3SA} *UAS-vg*^{TONDU} versus *esg*^{ts}>*yki*^{3SA} tumors (blue). (C) X-Y correlation plot for B.

Figure S5

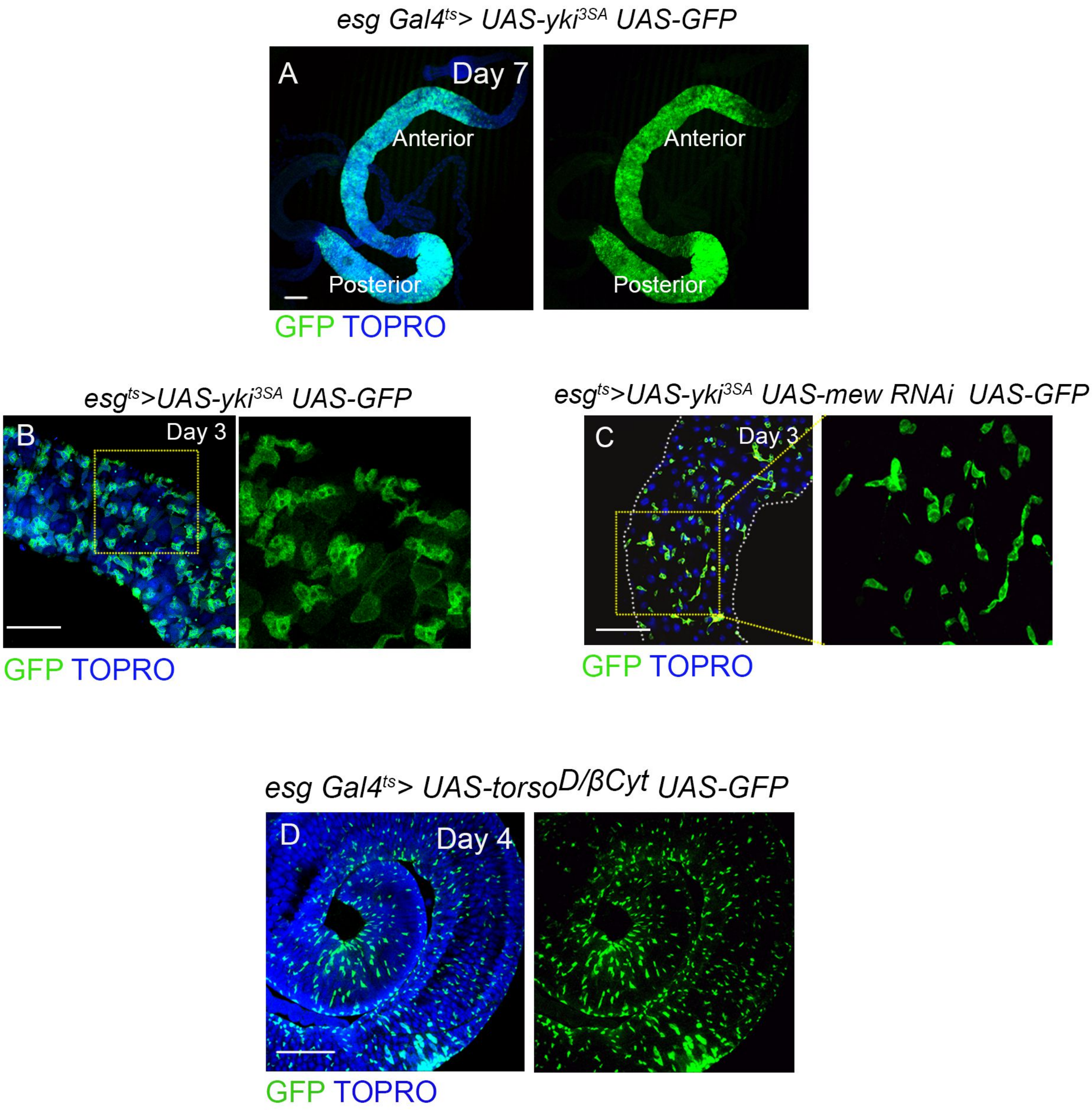

Figure S5. Loss of integrin signaling in Yki-driven ISC tumors. (A) *esg*^{ts}>*yki*^{3SA} tumors (day 7) control for *esg*^{ts}>*yki*^{3SA} *UAS-rhea RNAi* (see Fig. 4G). (B-C) Early *esg*^{ts}>*yki*^{3SA} *UAS-mew RNAi* tumors (day 3) display small ISC clusters (C) compared to similarly aged *esg*^{ts}>^{yki3SA} tumors (B). (D) Constitutive gain of integrin signaling in *esg*^{ts}>*UAS-torso*$^{D/\beta Cyt}$ as seen on day 4 of Gal4 activation. No aberrant increase in ISC numbers was observed. Scale bars 100 µm.

Disease Models & Mechanisms • Supplementary information

Table S1. List of proteins identied by unlabeled LC-MS/MS proteomic analysis of Yki-driven Intestinal Stem Cell (ISC) tumors in *Drosophila* adult midguts.

Click here to Download Table S1

Table S2. Proteins with significant fold change ($\geq\pm\log_2 2$, $p<0.05$) in day 7 versus day 1 of $esg^{ts}>yki^{3SA}$ driven ISC tumors.

Gene ID	Gene Symbol	Protein FDR Confidence: Combined	Master	UniProt Accession	Median *(Day 1)*	Median *(Day 7)*	Abundance Ratio: Day 7/ Day 1	Abundance Ratio (log2)	T-Test (P value)	# amino acis	MW [kDa]	# Peptides (SequestHT)	Chromosome	Found in Sample Group: (Day 1)	Found in Sample Group: (Day 7)
**FBgn0261276**	*Opa1*	High	None	F0JAH2	74046.703	297394.711	4.016	**2.006**	**0.0048**	453	51.2	2	2R	Peak Found	High
**FBgn0000562**	*egl*	Medium	Master Protein Candidate	Q9W1K4	14056.321	57460.557	4.088	**2.031**	**0.0430**	1004	112.1	1	2R	Peak Found	High
**FBgn0260442**	*rhea*	High	Master Protein	Q960C2	67243.947	276212.477	4.108	**2.038**	**0.0264**	1601	171.2	26	3L	High	High
**FBgn0035498**	*Fit1*	High	Master Protein	Q9VZI3	394737.461	1640060.666	4.155	**2.055**	**0.0276**	708	80.4	6	3L	High	High
**FBgn0035793**	*CG7546*	High	Master Protein Candidate	M9PBU3	91147.313	390343.234	4.283	**2.098**	**0.0087**	1179	125.7	2	3L	High	High
**FBgn0030955**	*CG6891*	High	Master Protein Candidate	Q8MQZ6	100332.746	448638.875	4.472	**2.161**	**0.0323**	269	30.2	1	X	High	High
**FBgn0013437**	*copia*	High	Master Protein	P04146	581703.875	2664281.188	4.580	**2.195**	**0.0138**	1409	162.7	3		High	High
**FBgn0265991**	*Zasp52*	High	None	G3JX29	147506.977	678663.543	4.601	**2.202**	**0.0366**	651	70.9	3	2R	High	High
**FBgn0015379**	*dod*	High	Master Protein	P54353	10240.124	94620.563	9.240	**3.208**	**0.0451**	166	18.4	1	X	Peak Found	High
**FBgn0000667**	*Actn*	High	Master Protein Candidate	M9MS06	60172.696	284884.955	4.734	**2.243**	**0.0489**	895	103.8	4	X	Peak Found	High
**FBgn0010434**	*cora*	High	Master Protein Candidate	A0A0B4 LFX4	469318.387	2248560.410	4.791	**2.260**	**0.0047**	1600	173.8	6	2R	High	High
**FBgn0014020**	*Rho1*	High	Master Protein	P48148	491985.963	2396139.059	4.870	**2.284**	**0.0177**	192	21.7	6	2R	Peak Found	High
**FBgn0030052**	*CG12065*	High	None	Q8MRM6	279747.601	1375997.625	4.919	**2.298**	**0.0460**	641	71.1	5	X	Peak Found	High
**FBgn0053470**	*CG33470; IM10; IMPPP*	High	Master Protein	Q8ML70	3874.029	38433.805	9.921	**3.310**	**0.0377**	257	28	1	2R	Peak Found	High
**FBgn0035499**	*Chd64*	High	None	M9PE30	6518473.696	32604473.558	5.002	**2.322**	**0.0003**	175	19.4	25	3L	High	High
**FBgn0025352**	*Mtpβ*	High	Master Protein	O77466	60337.865	605581.250	10.037	**3.327**	**0.0340**	469	50.6	1	2R	Peak Found	High
**FBgn0086346**	*ALiX*	High	Master Protein	Q9VB05	77987.759	397127.230	5.092	**2.348**	**0.0169**	836	92.5	2	3R	Peak Found	High
**FBgn0260442**	*rhea*	High	None	M9NDM3	1072195.987	5647432.310	5.267	**2.397**	**0.0350**	2169	235.1	31	3L	High	High
**FBgn0004397**	*Vinc*	High	Master Protein	X2JAB9	741152.635	3905646.674	5.270	**2.398**	**0.0224**	961	106.2	12	X	High	High
**FBgn0021760**	*chb*	High	Master Protein	Q9NBD7	31028.359	169383.656	5.459	**2.449**	**0.0329**	1491	165.5	2	3L	Peak Found	High
**FBgn0262567**	*CG43107*	High	Master Protein	D0IQC0	9607.135	107383.602	11.177	**3.483**	**0.0174**	63	6.8	1	2R	Peak Found	Peak Found
**FBgn0013733**	*shot*	High	Master Protein	A1Z9J3	28039.734	350253.043	12.491	**3.643**	**0.0138**	8805	988.9	2	2R	Peak Found	High
**FBgn0033446**	*CG1648*	High	Master Protein	Q7K2P3	775279.577	5030252.721	6.488	**2.698**	**0.0201**	230	23.8	18	2R	High	High
**FBgn0262735**	*Imp*	High	Master Protein	Q0KHU2	88808.606	580638.375	6.538	**2.709**	**0.0201**	631	69.4	3	X	Peak Found	High
**FBgn0022709**	*Adk1*	High	Master Protein Candidate	Q9VTV3	17311.554	113919.848	6.581	**2.718**	**0.0072**	201	21.9	1	3L	Peak Found	High
**FBgn0086906**	*sls*	High	None	R4UAY6	23315.416	308855.279	13.247	**3.728**	**0.0122**	662	74.6	2	3L	High	High
**FBgn0010909**	*msn*	Medium	None	Q7KV90	7461.706	102635.387	13.755	**3.782**	**0.0121**	1200	130.3	1	3L	Peak Found	High

Disease Models & Mechanisms • Supplementary information

**FBgn0036580**	*PDCD-5*	High	Master Protein	Q9VUZ8	11643.499	169815.949	14.585	**3.866**	**0.0111**	133	15.1	2	3L	Peak Found	Peak Found
**FBgn0051901**	*Mur29B*	Medium	Master Protein Candidate	Q8MS63	22220.121	166646.781	7.500	**2.907**	**0.0366**	339	35.7	1	2L	High	High
**FBgn0265434**	*zip*	High	None	A0A0B4JD95	3745790.734	28852286.631	7.703	**2.945**	**0.0010**	1964	226.6	67	2R	High	High
**FBgn0005666**	*bent*	High	None	O76281	284599.331	2219016.392	7.797	**2.963**	**0.0385**	6658	743	15	4	Peak Found	High
**FBgn0262716**	*Arp3; Arp66B*	High	Master Protein	P32392	91762.304	725246.529	7.904	**2.982**	**0.0397**	418	47	4	3L	High	High
**FBgn0053113**	*Rtnl1*	High	Master Protein Candidate	Q9VMV9	594076.854	5048772.010	8.499	**3.087**	**0.0238**	595	63.9	9	2L	High	High
**FBgn0085282**	*CG34253*	High	Master Protein	A8JNV2	26812.808	228363.336	8.517	**3.090**	**0.0221**	112	12.5	1	3L	Peak Found	High
**FBgn0265434**	*zip*	High	None	J7JVR0	1687952.657	14541350.003	8.615	**3.107**	**0.0003**	1425	164.4	39	2R	High	High
**FBgn0016726**	*RpL29*	High	Master Protein	B7FNL1	732637.288	6371885.220	8.697	**3.121**	**0.0452**	85	10	6	2R	High	High
**FBgn0029766**	*CG15784*	High	Master Protein	Q9W4C1	151556.702	1363172.164	8.994	**3.169**	**0.0261**	554	62.3	5	X	High	High
**FBgn0038774**	*CG5023*	High	Master Protein	Q9I7J0	580277.645	5744084.078	9.899	**3.307**	**0.0314**	169	19.1	9	3R	High	High
**FBgn0001217**	*Hsc70-2*	High	Master Protein	P11146	34573.184	694860.797	20.098	**4.329**	**0.0135**	633	69.7	3	3R	Peak Found	High
**FBgn0051363**	*CG31363*	High	Master Protein	B5RJ67	30460.900	612434.938	20.106	**4.330**	**0.0112**	230	24.4	1	3R	Peak Found	High
**FBgn0000043**	*Act42A*	High	Master Protein	P02572	13317.133	326000.129	24.480	**4.614**	**0.0155**	376	41.8	37	2R	Peak Found	High
**FBgn0004227**	*nonA*	High	Master Protein	Q8IR16	51621.380	664389.951	12.870	**3.686**	**0.0092**	742	81.9	5	X	High	High
**FBgn0011225**	*jar*	High	Master Protein	Q01989	32407.746	421589.398	13.009	**3.701**	**0.0170**	1253	143.2	6	3R	Peak Found	High
**FBgn0034709**	*CG3074; Swim*	High	Master Protein	Q7JWQ7	582800.990	7699009.721	13.210	**3.724**	**0.0014**	431	48.8	11	2R	High	High
**FBgn0031436**	*CG3214; ND-B17.2*	High	Master Protein	Q9VQD7	13005.392	404752.090	31.122	**4.960**	**0.0032**	142	16.8	2	2L	High	High
**FBgn0000639**	*Fbp1*	High	Master Protein Candidate	M9PFK6	2941545.182	75592.226	0.026	**-5.282**	**0.0184**	1027	119.4	15	3L	High	Peak Found
**FBgn0033297**	*Mal-A8*	High	Master Protein Candidate	H5V882	145483.008	4449.611	0.031	**-5.031**	**0.0020**	597	68.1	1	2R	High	Peak Found
**FBgn0010497**	*dmGlut; l(2)01810*	Medium	Master Protein Candidate	Q95R95	217644.070	7972.730	0.037	**-4.771**	**0.0143**	165	17.7	1	2L	High	Peak Found
**FBgn0004426**	*LysC*	High	None	P83971	1020142.977	101441.592	0.099	**-3.330**	**0.0171**	140	15.6	2	3L	High	Peak Found
**FBgn0031141**	*CG1304*	High	None	Q9VRD1	1379758.828	251684.488	0.182	**-2.455**	**0.0240**	260	27.8	1	X	High	High
**FBgn0004427**	*LysD*	High	Master Protein Candidate	P83972	2366383.414	523926.295	0.221	**-2.175**	**0.0126**	140	15.6	3	3L	High	High
**FBgn0004425**	*LysB*	High	Master Protein Candidate	Q08694	2366383.414	523926.295	0.221	**-2.175**	**0.0126**	140	15.6	3	3L	High	High
**FBgn0004428**	*LysE*	High	Master Protein	P37159	2366383.414	523926.295	0.221	**-2.175**	**0.0126**	140	15.5	3	3L	High	High
**FBgn0034662**	*CG13492*	High	Master Protein	Q8MLU9	1715613.215	393704.303	0.229	**-2.124**	**0.0428**	2979	321.1	12	2R	High	High
**FBgn0040349**	*CG3699*	High	Master Protein Candidate	Q9U1L2	6207241.123	1490634.446	0.240	**-2.058**	**0.0024**	251	26	12	X	High	High

Disease Models & Mechanisms • Supplementary information

Table S3. Interacting partners belonging to the Hippo pathway for proteins with ≥log2 2 fold change in *esg*^{ts}>*yki*^{3SA} driven ISC tumors.

Protein	Fold change (log_2 fold)	T Test P value		Uniprot	[$]Hippo protein-protein interaction network	
					Protein-and-Hippo pathway member	Score
Cora	2.2604	0.0047	High	A0A0B4LFX4	Cora ←→Ft	1*
Mtb-β	3.3272	0.0340	Peak Found	O77466	Mtb-β ←→Ft	0.99*
Msn	3.7819	0.0121	Peak Found	Q7KV90	Msn ←→Ft	1*
nonA	3.6860	0.0092	High	Q8IR16	nonA ←→Ex	0.82*
Jar	3.7014	0.0170	Peak Found	Q01989	Jar ←→Wts	0.89*
Talin	2.0383	0.0264	High	Q960C2	Talin ←→Yki	0.33
Vinc	2.2184	0.0188	High	Q24584	Vinc ←→Ft	0.36
Chd64	2.3225	0.0003	High	M9PE30	Chd64 ←→Yki	0.01
Mtb-β	3.3272	0.0340	Peak Found	O77466	Mtb-β ←→Wts	0.1
Arp3	2.9825	0.0397	High	P32392	Arp3 ←→Wts	0.21
Rtnl1	3.0872	0.0238	High	Q9VMV9	Rtnl1 ←→Ft	0.38
nonA	3.6860	0.0092	High	Q8IR16	nonA ←→Wts	0.28

[$]Kwon *et al.* (Kwon et al., 2013). *Statistically significant intections

Table S4. Gene Ontology (GO) analysis of enriched proteins in *esg*^{ts}>*yki*^{3SA} driven ISC tumors.

MOLECULAR FUNCTION		[$]GO classes	Gene Numbers	% representation	% representation
	1	binding (GO:0005488)	7	19.40%	29.20%
	2	structural molecule activity (GO:0005198)	6	16.70%	25.00%
	3	molecular function regulator (GO:0098772)	1	2.80%	4.20%
	4	catalytic activity (GO:0003824)	10	27.80%	41.70%
**BIOLOGICAL PROCESS**					
	1	response to stimulus (GO:0050896)	2	5.60%	7.40%
	2	cellular process (GO:0009987)	11	30.60%	40.70%
	3	multicellular organismal process (GO:0032501)	1	2.80%	3.70%
	4	metabolic process (GO:0008152)	6	16.70%	22.20%
	5	biological regulation (GO:0065007)	2	5.60%	7.40%
	6	localization (GO:0051179)	4	11.10%	14.80%
	7	biological adhesion (GO:0022610)	1	2.80%	3.70%
**CELLULAR COMPONENT**					
	1	organelle (GO:0043226)	9	25.00%	45.00%
	2	extracellular region (GO:0005576)	1	2.80%	5.00%
	3	cell (GO:0005623)	10	27.80%	50.00%
**PROTEIN CLASSES**					
	1	transmembrane receptor regulatory/adaptor protein (PC00226)	1	2.80%	4.20%
	2	hydrolase (PC00121)	5	13.90%	20.80%
	3	cell junction protein (PC00070)	1	2.80%	4.20%

Disease Models & Mechanisms • Supplementary information

	4	enzyme modulator (PC00095)	6	16.70%	25.00%
	5	nucleic acid binding (PC00171)	3	8.30%	12.50%
	6	transferase (PC00220)	1	2.80%	4.20%
	7	receptor (PC00197)	1	2.80%	4.20%
	8	cytoskeletal protein (PC00085)	5	13.90%	20.80%
	9	structural protein (PC00211)	1	2.80%	4.20%
**PATHWAYS**					
	1	Gonadotropin-releasing hormone receptor pathway (P06664)	1	2.80%	4.30%
	2	Cadherin signaling pathway (P00012)	1	2.80%	4.30%
	3	De novo purine biosynthesis (P02738)	1	2.80%	4.30%
	4	Axon guidance mediated by Slit/Robo (P00008)	1	2.80%	4.30%
	5	Apoptosis signaling pathway (P00006)	1	2.80%	4.30%
	6	Integrin signalling pathway (P00034)	5	13.90%	21.70%
	7	Angiogenesis (P00005)	1	2.80%	4.30%
	8	Alzheimer disease-presenilin pathway (P00004)	1	2.80%	4.30%
	9	Inflammation mediated by chemokine and cytokine signaling pathway (P00031)	2	5.60%	8.70%
	10	Huntington disease (P00029)	1	2.80%	4.30%
	11	Parkinson disease (P00049)	1	2.80%	4.30%
	12	Ras Pathway (P04393)	1	2.80%	4.30%
	13	Cytoskeletal regulation by Rho GTPase (P00016)	3	8.30%	13.00%
	14	Nicotinic acetylcholine receptor signaling pathway (P00044)	3	8.30%	13.00%

$GO enrichment was determined using PANTHER (http://www.pantherdb.org/).

Table S5. Status of integrin pathway members in *esg*^{ts}>*yki*^{3SA} driven ISC tumors.

**Gene ID**	**Pathway member**	**Fly base ID**	**Proteomics** (log_2 fold). Current study	**$RNAseq** ($log_2$ fold)
scb/aPS3	Integrin receptor	FBgn0003328	2.6069 (UP, P=0.270)	3.2797 (UP)
rhea	Adaptor	FBgn0260442	2.3970 (UP, P=0.035)	2.4646 (UP)
Ilk	Kinase and Scaffold protein	FBgn0028427	2.0724 (UP, P=0.056)	1.6871 (UP)
Pax	Scaffold protein	FBgn0041789	6.0551 (UP, P=0.051)	1.6507 (UP)
Vinc	Scaffold protein	FBgn0004397	2.3977 (UP, P=0.022)	2.8706 (UP)
vkg	Basemen Membrane	FBgn0016075	1.8255 (UP, P=0.014)	2.5527 (UP)
Rho1	GTPase	FBgn0014020	2.2840 (UP, P=0.018)	2.3794 (UP)
Act42A	Cytoskeleton	FBgn0000043	4.6013 (UP, P=0.1934)	5.1188 (UP)
mew/alpha-PS1	Integrin receptor	FBgn0004456	not found	3.3184 (UP)
mys/betaPS1	Integrin receptor	FBgn0004657	not found	3.7469 (UP)
if/alphaPS2,	Integrin receptor	FBgn0001250	not found	0.3507
Itgbn/Itgbetanu	Integrin receptor	FBgn0010395	not found	0.1434
LanA	Ligand	FBgn0002526	not found	3.0021 (UP)
wb/LanA1	Ligand	FBgn0261563	not found	2.0211 (UP)
LanB1	Ligand	FBgn0261800	not found	2.4286 (UP)

$ Song *et al.* (Song et al., 2019).

Disease Models & Mechanisms • Supplementary information

Table S6. Putative Yki-Sd binding sites[$] in regulatory regions of genes found enriched in *esg*^{ts}>*yki*^{3SA} tumors.

Fly base ID	Gene Symbol	Fold enrichment in *esg*^{ts}>*yki*^{3SA} tumors	Chromosome	[$]Yki-Sd binding site from TSS
FBgn0261276	*opa1*	2.0059	2R	-690*
FBgn0000562	*egl*	2.0314	2R	-9.5
FBgn0035498	*Fit1*	2.0548	3L	-317
FBgn0035793	*CG7546*	2.0985	3L	-533, 193
FBgn0030955	*CG6891*	2.1608	X	-682; -316; 456
FBgn0015379	*dod*	3.2079	X	-29.5
FBgn0000667	*Actn*	2.2432	X	233.5
FBgn0010434	*cora*	2.2604	2R	346.5
FBgn0014020	*Rho1*	2.2840	2R	22.5; 1058.5
FBgn0030052	*CG12065*	2.2983	X	87; 647.5
FBgn0035499	*Chd64*	2.3225	3L	57.5
FBgn0025352	*Mtpbeta*	3.3272	2R	-129
FBgn0021760	*chb*	2.4486	3L	-100; 92; 841
FBgn0013733	*shot*	3.6429	2R	94; -421.5; -1118.5; 7211; 839; 503; -114.5
FBgn0033446	*CG1648*	2.6978	2R	1867
FBgn0010909	*msn*	3.7819	3L	446; -30.5
FBgn0053113	*Rtnl1*	3.0872	2L	-599.5; -308.5; -1035.5; 90.5
FBgn0016726	*Rpl-29B*	3.1205	2R	-77
FBgn0000043	*Act42A*	4.6135	2R	-52; -365
FBgn0011225	*jar*	3.7014	3R	-562.5
FBgn0028427	*ilk*	2.0724	3L	-4.5
FBgn0041789	*Pax*	6.0551	2L	-19
FBgn0004397	*vinc*	2.3977	X	64.5; -614
FBgn0004456	*mew*	not found	X	-185
FBgn0004657	*alphaPS2*	not found	X	630.5; 1586; 1995.5
FBgn0010395	*Itgbeta nu*	not found	2L	-110
FBgn0002526	*lanA*	not found	3L	-569.5; -1244

[$]Nagaraj *et al.* (Nagaraj et al., 2012).

Table S7. Change in levels of proteins in TONDU peptide-treated *esg*^{ts}>*yki*^{3SA} driven ISC tumors.

				TREATED	
Fly base ID	Gene symbol	FDR confidence	UniProt Accession	Abundance Ratio.	T-Test (*p* value)
FBgn0261276	*opa1*	High	F0JAH2	-0.9677	0.0612
FBgn0000562	*egl*	Medium	Q9W1K4	Not found	Not found
FBgn0260442	*rhea*	High	Q960C2	-0.9850	0.6182
FBgn0035498	*Fit1*	High	Q9VZI3	-1.3108	0.4323
FBgn0035793	*CG7546*	High	M9PBU3	-1.9655	0.0057
FBgn0030955	*CG6891*	High	Q8MQZ6	-2.5940	0.0034
FBgn0013437	*GIP*	High	P04146	-2.1634	0.0034
FBgn0265991	*Zasp52*	High	G3JX29	-1.2146	0.1208
FBgn0015379	*dod*	High	P54353	not found	Not found
FBgn0000667	*Actn*	High	M9MS06	-1.3641	0.0427
FBgn0010434	*cora*	High	A0A0B4LFX4	-1.0595	0.0051
FBgn0014020	*Rho1*	High	P48148	-0.8551	0.0536
FBgn0030052	*CG12065*	High	Q8MRM6	-1.5401	0.0876

FBgn0053470	*CG33470; IM10; IMPPP*	High	Q8ML70	-0.7563	0.4986
FBgn0035499	*Chd64*	High	M9PE30	-1.2033	0.0076
FBgn0025352	*Mtpbeta*	High	O77466	-2.3300	0.0139
FBgn0086346	*ALiX*	High	Q9VB05	-2.3246	0.0151
FBgn0260442	*rhea*	High	M9NDM3	-1.2592	0.0478
FBgn0004397	*Vinc*	High	X2JAB9	-1.3361	0.0365
FBgn0021760	*chb*	High	Q9NBD7	-2.1663	0.0263
FBgn0262567	*CG43107*	High	D0IQC0	-1.6068	0.1094
FBgn0013733	*shot*	High	A1Z9J3	not found	Not found
FBgn0033446	*CG1648*	High	Q7K2P3	-1.2957	0.0316
FBgn0262735	*Imp*	High	Q0KHU2	-1.6114	0.0313
FBgn0022709	*Adk1*	High	Q9VTV3	-1.5700	0.0068
FBgn0086906	*sls*	High	R4UAY6	-1.2668	0.0439
FBgn0010909	*msn*	Medium	Q7KV90	-0.9091	0.0273
FBgn0036580	*PDCD-5*	High	Q9VUZ8	-1.1028	0.3096
FBgn0051901	*Mur29B*	Medium	Q8MS63	-1.0776	0.0244
FBgn0265434	*zip*	High	A0A0B4JD95	-1.1648	0.0140
FBgn0005666	*bt*	High	O76281	-2.5538	0.0038
FBgn0262716	*Arp3; Arp66B*	High	P32392	-1.1503	0.0234
FBgn0053113	*Rtnl1*	High	Q9VMV9	-1.2851	0.0129
FBgn0085282	*CG34253*	High	A8JNV2	-0.4406	0.0846
FBgn0265434	*zip-RC*	High	J7JVR0	-1.1466	0.0063
FBgn0016726	*Rpl-29B*	High	B7FNL1	-1.8229	0.1047
FBgn0029766	*CG15784*	High	Q9W4C1	-2.6531	0.0395
FBgn0038774	*CG5023*	High	Q9I7J0	-1.3464	0.0059
FBgn0001217	*Hsc70-2*	High	P11146	-1.5196	0.0343
FBgn0051363	*Jupiter-RD*	High	B5RJ67	not found	not found
FBgn0000043	*Act42A*	High	P02572	-3.6540	0.0297
FBgn0004227	*nonA*	High	Q8IR16	-0.8528	0.0079
FBgn0011225	*jar*	High	Q01989	-0.3926	0.4731
FBgn0034709	*CG3074; Swim*	High	Q7JWQ7	-1.6677	0.0054
FBgn0031436	*CG3214; ND-B17.2*	High	Q9VQD7	-0.3077	0.4512

Disease Models & Mechanisms • Supplementary information

Table S8. Fly lines and their source.

	Fly Genotype	Source	
1	*UAS-yki*$^{S111A.S168A.S250A}$	#BDSC	#28817
2	*UAS-mew RNAi*	#BDSC	#27543
3	*UAS-rhea RNAi*	#BDSC	#28950
4	*lgl*^{4}*FRT 40A*	#BDSC	#36289
5	*esg-Gal4*	Norbert Perrimon, Harvard Medical School, USA	-
6	*UAS–torso*^{D}*/βcyt*	Nick Brown, University of Cambridge, UK	-

#Bloomington Drosophila Stock Center, Indiana University, Bloomington.

Table S9. List of antibodies their source and working dilutions.

	Protein	Catalog Number	Source	Raised in	Working dilution
1	Delta (extra cellular domain)	C594.9B	#DSHB	mouse	1:50
2	Sd	-	Gift from (De Celis lab)	rabbit	1:100
3	Talin (carboxy terminus 534 amino acids)	A22A	#DSHB	mouse	1:100
4	αPS1 , Mew	DK.1A4	#DSHB	mouse	1:50

#Developmental Studies Hybridoma Bank, University of Iowa.

Table S10. List of PCR primers.

		Forward primer	Reverse primer
	**For expression analysis**		
1.	*ImpL2*	AAGAGCCGTGGACCTGGTA	TTGGTGAACTTGAGCCAGTCG
2.	*yki*	CCTTGCCGCCGGGATGG	TTTGCTGCTGCTGGCGATATTG
3	*delta*	AGCGACTCTTGGTGCAGCAGGTACT	TCCGTAGTAGTTGAGATCGCAGGTGAC
4	*myc*	ACACGCGCTGCAACGATATGG	CGAGGGATTTGTGGGTAGCTTCTT
5	*wg*	TGATGGCCCTGTGCAGCG	TGATGGCCCTGTGCAGCG
6	*ex*	GCCGCCTTTACCTGTCCAAC	CGTTCCGGTTTCCAATTAGCT
7	*β-tub*	CAAGCTGGCAGTGCGGCAAC	GCTGTCACCGTGGTAGGCGCC
8	*YAP1*	ACGTTCATCTGGGACAGCAT	GTTGGGAGATGGCAAAGACA
9	*GAPDH*	TGCACCACCAACTGCTTAGC	GGCATGGACTGTGGTCATGAG
	**ChIP PCR**		
10	*Mew*	GCTTTGGTGGGGCTTGTAAC	GTAAAGGCATGAGCGCCAAAT

Disease Models & Mechanisms • Supplementary information

Table S11. Genotype of the *Drosophila* lines used in this study

	Description	Figures	Genotype
1	Control flies bearing GFP-marked ISCs.	Pertaining to figures 1 and 4	*esg-Gal4, tub-Gal80^{ts}, UAS-GFP; +*
2	Gain of constitutively active Yki in ISCs	Pertaining to figures 1, 2 and 4; figures S1 and S3	*esg-Gal4, tub-Gal80^{ts}, UAS-GFP/+; UAS-Yki^{3SA}/+* (*UAS-yki$^{S111A.S168A.S250A}$* referred to as *UAS-Yki^{3SA}*)
3	Simultaneous gain of constitutively active Yki (*UAS-Yki^{3SA}*) and TONDU peptide in ISCs.	Pertaining to figure 2; figure S2	*esg-Gal4, tub-Gal80^{ts}, UAS-GFP/+; UAS-Yki^{3SA}/+UAS-vg^{TONDU}/+*
4	Downregulation of *mew* in ISCs expressing a constitutively active Yki	Pertaining to figure 4	*esg-Gal4, tub-Gal80^{ts}, UAS-GFP/+; UAS-Yki^{3SA}/+UAS-mew RNAi/+*
5	Downregulation of *rhea* in ISCs expressing a constitutively active Yki	Pertaining to figure S5	*esg-Gal4, tub-Gal80^{ts}, UAS-GFP/+; UAS-Yki^{3SA}/+ UAS-rhea RNAi/+*
6	Gain of constitutively active βintegrin in ISCs.	Pertaining to figure S5	*esg-Gal4, tub-Gal80^{ts}, UAS-GFP/+; UAS–torso^{D}/βcyt*

REFERENCES

Kwon, Y., Vinayagam, A., Sun, X., Dephoure, N., Gygi, S. P., Hong, P. and Perrimon, N. (2013). The Hippo signaling pathway interactome. *Science* **342**, 737-40.

Nagaraj, R., Gururaja-Rao, S., Jones, K. T., Slattery, M., Negre, N., Braas, D., Christofk, H., White, K. P., Mann, R. and Banerjee, U. (2012). Control of mitochondrial structure and function by the Yorkie/YAP oncogenic pathway. *Genes Dev* **26**, 2027-37.

Song, W., Kir, S., Hong, S., Hu, Y., Wang, X., Binari, R., Tang, H. W., Chung, V., Banks, A. S., Spiegelman, B. et al. (2019). Tumor-Derived Ligands Trigger Tumor Growth and Host Wasting via Differential MEK Activation. *Dev Cell* **48**, 277-286 e6.
